# The Complete Chloroplast Genomes of *Primula obconica* Provide Insight That Neither Species nor Natural Section Represent Monophyletic Taxa in *Primula* (Primulaceae)

**DOI:** 10.3390/genes13040567

**Published:** 2022-03-23

**Authors:** Qiang Li

**Affiliations:** Key Laboratory of Adaptation and Evolution of Plateau Biota, Northwest Institute of Plateau Biology, Chinese Academy of Sciences, Xining 810008, China; liqiang9503@163.com

**Keywords:** *Primula obconica*, *Primula*, chloroplast genome, intraspecific variation, phylogenetic

## Abstract

The genus *Primula* (Primulaceae) comprises more than 500 species, with 300 species distributed in China. The contradictory results between systematic analyses and morphology-based taxonomy make taxonomy studies difficult. Furthermore, frequent introgression between closely related species of *Primula* can result in non-monophyletic species. In this study, the complete chloroplast genome of sixteen *Primula obconica* subsp. *obconica* individuals were assembled and compared with 84 accessions of 74 species from 21 sections of the 24 sections of the genus in China. The plastome sizes of *P. obconica* subsp. *obconica* range from 153,584 bp to 154,028 bp. Genome-wide variations were detected, and 1915 high-quality SNPs and 346 InDels were found. Most SNPs were detected in downstream and upstream gene regions (45.549% and 41.91%). Two cultivated accessions, ZP1 and ZP2, were abundant with SSRs. Moreover, 12 SSRs shared by 9 accessions showed variations that may be used as molecular markers for population genetic studies. The phylogenetic tree showed that *P. obconica* subsp. *obconica* cluster into two independent clades. Two subspecies have highly recognizable morphological characteristics, isolated geographical distribution areas, and distinct phylogenetic relationships compared with *P. obconica* subsp. *obconica*. We elevate the two subspecies of *P. obconica* to separate species. Our phylogenetic tree is largely inconsistent with morphology-based taxonomy. Twenty-one sections of *Primula* were mainly divided into three clades. The monophyly of Sect. *Auganthus*, Sect. *Minutissimae*, Sect. *Sikkimensis*, Sect. *Petiolares*, and Sect. *Ranunculoides* are well supported in the phylogenetic tree. The Sect. *Obconicolisteri*, Sect. *Monocarpicae*, Sect. *Carolinella*, Sect. *Cortusoides*, Sect. *Aleuritia*, Sect. *Denticulata*, Sect. *Proliferae Pax*, and Sect. *Crystallophlomis* are not a monophyletic group. The possible explanations for non-monophyly may be hybridization, polyploidization, recent introgression, incorrect taxonomy, or chloroplast capture. Multiple genomic data and population genetic studies are therefore needed to reveal the evolutionary history of *Primula*. Our results provided valuable information for intraspecific variation and phylogenetic relationships within *Primula*.

## 1. Introduction

Species are fundamental units of biodiversity, but the boundaries of species are still confusing [1], especially in species-rich lineages that share significant similarities, morphological characteristics, and ecological regions. Generally, prior species delimitation is based on morphological traits, which may fail to discriminate polymorphic taxa [2]. Due to the rapid development of next-generation sequencing technologies, chloroplast genomes have become easier to obtain. As “super-barcodes”, chloroplast genomes have been successful in solving taxonomic issues of closely related plant taxonomy [3], such as in *Ottelia acuminata*, which consists of six phenotypic varieties, of which the chloroplast genome was successfully divided it into two conspecific varieties [4], thus providing new insight into the use of the chloroplast genome as a super-barcode for species delimitation in taxonomically difficult plant taxa.

Intraspecies genetic and phenotypic variation is essential to drive plant evolution and adaptation [5], whereas organelle variation at the intraspecific level has been reported relatively rarely. Although the chloroplast genome in land plants has a typical and conservative genome structure, plastome structural variations occur both at the inter-and intraspecific levels. The length of cp genomes, IR contraction/expansion, gene length, and content at the intraspecific level (between the wild and cultivated species) are slightly different [6,7]. Moreover, the chloroplast genome possesses the properties of uniparental inheritance and a low frequency of recombination [8], which are widely used in phylogenetic investigation and species delimitation.

The genus *Primula* (Primulaceae) includes more than 500 species, with 300 species distributed in China [9]. The center of distribution of *Primula* was found in China (western Sichuan, northwestern Yunan, and eastern Xizang). From the morphological evidence, it was generally accepted that there are seven subgenera worldwide (subgen. *Sphondylia*, subgen. *Auriculastrum*, subgen. *Primula*, subgen. *Auganthus*, subgen. *Carolinella*, subgen. *Craibia*, and subgen. *Aleuritia*). However, this assumption was inconsistent with molecular phylogenetic analyses, because most of these subgenera were found to be non-monophyletic [10,11]. Furthermore, a large number of new species based exclusively on morphology have been published recently; the treatment of these new species has been based on morphology-based taxonomy [12,13,14]. China comprises twenty-five sections and the subgenus concept was not adopted. However, only a few sections were supported as monophyletic, such as Sect. *Auricula* [15], Sect. *Proliferae*, Sect. *Amethystina*, Sect. *Petiolares*, Sect. *Muscarioides*, Sect. *Souliei*, Sect. *Sikkimensis*, Sect. *Soldanelloides*, and Sect. *Pycnoloba* [16].

Heterostylous and homostylous taxa both occur in *Primula*, including within the same species (*P. obconica* and *P. oreodoxa*). Species such as *P. obconica* subsp. *obconica* are heterostylous, whereas species such as *P. obconica* subsp. *parva* are homostylous [17,18]. The vast majority of this genus is characterized by heterostyly. Moreover, heterostyly plays an important role in promoting outcrossing [19]. For this reason, interspecific hybridization is not rare in the genus *Primula*, such as *P. veris* × *P. vulgaris* and *P. elatior* × *P. vulgaris* [20]. Hybridization has been singularly important to the evolution and speciation of *Primula* [21], which may confuse the interspecific boundaries and bring difficulty to species delimitation, as well as exploration of the evolutionary history of the genus.

*Primula obconica* is widely distributed in subtropical China. It contains six subspecies, including *P. obconica* subsp. *obconica*, *P. obconica* subsp. *werringtonensis*, *P. obconica* subsp. *parva*, *P. obconica* subsp. *begoniiformis*, *P. obconica* subsp. *nigroglandulosa*, and *P. obconica* subsp. *fujianensis* [9,22]. These subspecies, except for *P. obconica* subsp. *obconica*, all have restricted areas of distribution in the Yunnan, Sichuan, and Fujian provinces of China. *P. obconica* shows a high variation in morphological traits at the intraspecific level, mainly in plant size and leaf blade indumentum [23]. Due to its high intraspecific characteristic variation, the taxonomy of *P. obconica* was confused, for example, *P. obconica* subsp. *werringtonensis*, as recognized by Smith, was treated as a separate species, *P. werringtonensis*. In addition, *P. obconica* subsp. *parva* has been treated as a separate species of *P. parva* [23,24]. Since its introduction to Europe in 1880, *P. obconica* subsp. *obconica* has been widely cultivated in Europe and North America [25]. Primin and miconidin, two allergic compounds that mainly cause skin hypersensitivity reactions, have been found in cultivated *P. obconica* subsp. *obconica*, but were not detected in wild species [26,27]. Wild germplasm may play an important role in guiding cultivated *Primula* breeding in the horticultural market [28].

The accurate delimitation of species boundaries is crucial for nature conservation and management. In rapidly evolved genera, hybridization and introgression are common, which enhance the difficulty of phylogenetic analyses. Furthermore, molecular systematic studies are inconsistent with the morphology-based taxonomy of *Primula*. Chloroplast genomes are widely used in phylogenetics, thus providing a solution for solving the evolutionary history of *Primula*. In this study, we analyzed a total of 100 accessions of 74 species from 21 sections of the 24 sections of the genus in China. Our main goals were to: (1) explore the intraspecific variation in *P. obconica* subsp. *obconica*; (2) decipher the taxonomic delimitation of the *P. obconica* complex; (3) reveal the phylogenetic relationships of 21 sections of *Primula* in China and test morphology-based taxonomy with molecular data.

## 2. Materials and Methods

### 2.1. Sample Collection, DNA Extraction, and Sequencing

Thirteen wild accessions were collected from the Sichuan, Yunnan, Hubei, and Hunan provinces. Samples were collected across China, including the main distribution areas of the species. Sichuan, Hubei, and Hunan included two populations, and each population contained two wild accessions. Two Chinese-cultivated accessions were collected from the Wuhu (Anhui) flower market (Table S1). All specimens were deposited in the Qinghai-Tibetan Plateau Museum of Biology, Northwest Institute of Plateau Biology, Chinese Academy of Sciences (HNWP). The silica gel-dried leaves were sent to Genepioneer Biotechnologies (Nanjing, China). DNA was extracted using a plant genomic DNA Kit (DP305), and paired-end reads of 2 × 150 bp for all samples were sequenced using NovaSeq 6000 (Illumina Inc., San Diego, CA, USA). The clean data were from 1.38 to 2.93 GB.

### 2.2. Genome Assembly and Annotation

The de novo assembly of the clean data was performed on a GetOrganelle pipeline [29], with -t 1, -R 10, using the *P. obconica* subsp. *obconica* (MK344754) plastome as a reference. Bandage was used to confirm that the assembly graph was circular [30]. Assembled genomes were annotated using CPGAVAS2 [31]. OrganellarGenomeDRAW version 1.3.1 was used to visualize the plastomes of *P. obconica* [32].

### 2.3. Reference Mapping, Variants Calling, and SSR Analysis

The complete chloroplast genome of *P. obconica* subsp. *obconica* (MK344754), was used as a reference. The Burrows–Wheeler Aligner (BWA) version 0.7.17 was used to index and align the clean data to the reference chloroplast genome [33], SAMtools version 1.9 was used to convert and sort the mapping results [34], SNP and InDel calling were filtered out (QUAL < 60.0, DP > 20, QD < 1.5) using GATK4 (with -ploidy 1) [35]. The program SnpEff version 4 was used to annotate and predict the effects of SNPs in *P. obconica* [36]. To identify the difference in simple sequence repeats (SSRs) between wild and cultivated *P. obconica*, the microsatellite identification (MISA) software tool was used, with 10, 6, 5, 3, 3, and 3 for mono-, di-, tri-, tetra-, penta-, and hexanucleotide sequences, respectively [37].

### 2.4. Comparative Analysis of the Chloroplast Genomes

A comparative analysis of the chloroplast genomes of sixteen wild and cultivated accessions was carried out using mVISTA online software (https://genome.lbl.gov/vista/mvista/submit.shtml, accessed on 30 August 2021) with a shuffle-LAGAN model, and HBD1 as a reference. To compare the IR/SC boundaries of different subspecies within *P. obconica*, the IRscope web service (https://irscope.shinyapps.io/irapp, accessed on 7 September 2021) was used to visualize the result.

### 2.5. Population Structure Analysis of P. obconica subsp. obconica

From the SNPs and InDels used in the aforementioned step, 1361 SNPs and 321 InDels were analyzed after filtering with the parameters mac 3 and max-missing 0.5. Sixteen sequenced accessions were converted into a concatenated sequence in Fasta format using PGDSpider version 2.1.1.5 [38]. The ML tree based on SNPs was constructed by IQ-TREE version 2 [39], with the K3P model. PCA was conducted by Plink version 1.9 [40]. Gene flow was inferred using TreeMix version 1.12 [41].

### 2.6. Phylogenetic Analysis

To study the relationships between *Primula*, we used 100 accessions of 74 species from 21 sections of the 24 sections of the genus *Primula* in China. An ML phylogenetic tree was performed, using *Pentaphylax euryoides* (MF179498) as an outgroup. Seventy-seven protein-coding genes were extracted, aligned, and merged by PhyloSuit version 1.1.2 [42]. Maximum likelihood (ML) estimation with 1000 bootstrap replications was conducted using IQ-TREE version 2 [39], with a TVM + F + R3 model from the result of ModelFinder.

## 3. Results

### 3.1. General Features of the P. obconica subsp. obconica Chloroplast Genome

**Figure 1:**
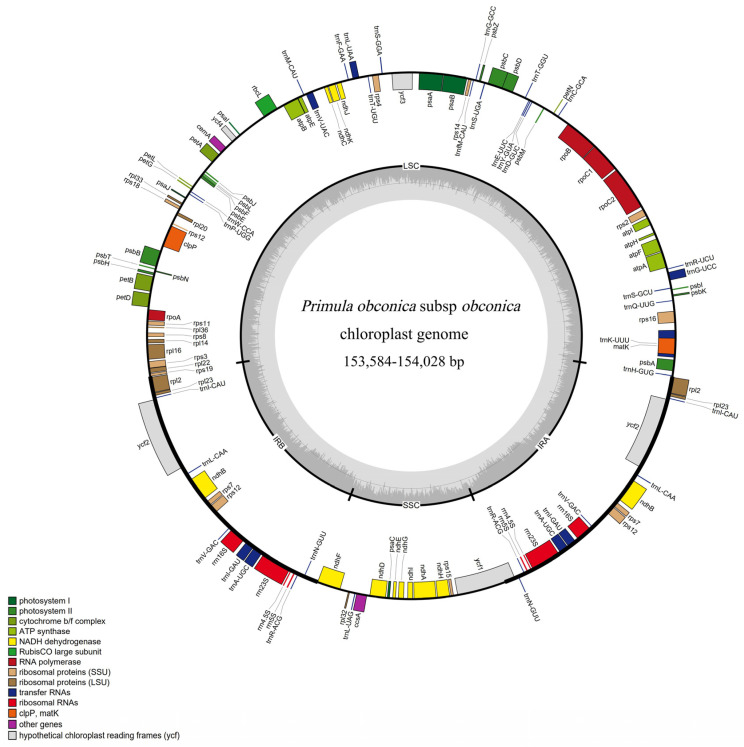
Chloroplast genome map of *P. obconica* subsp. *obconica*.

The 16 accessions of *P. obconica* subsp. *obconica* had a conventional quadripartite structure characteristic of angiosperm plastomes, with an LSC, SSC, and two IR regions (Figure 1). The chloroplast genomes ranged from 153,584 bp (SCB1) to 154,028 bp (HNL2 and HND1). The LSC and SSC regions have a relatively higher length variation compared with the IR regions, for example, the length of the LSC region was 84,712 bp and 84,403 bp in SCD1 and YN1 chloroplast genomes, respectively. The SSC region ranged from 17,743 bp (HNL2, HND1) to 17,347 bp (SCD1) (Table S2). A total of 128 genes were detected, including 83 protein-coding genes, eight ribosomal RNAs, and 37 transfer RNAs (Table S3). There were six protein-coding genes with the duplication genes and 22 genes that contained one intron. Two of these genes (*ycf3* and *clpP*) contained two introns. The *ycf3* and *clpP* genes were also trans-spliced into three exons (Table S4).

### 3.2. Chloroplast Genomic SNP Variations

**Figure 2:**
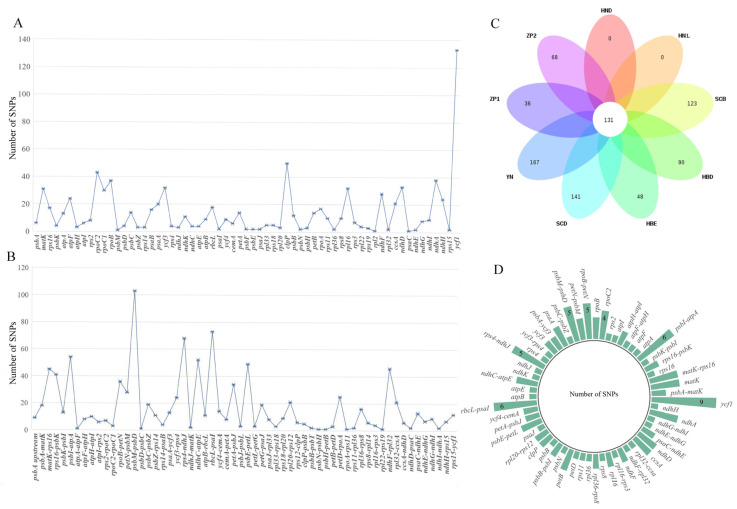
Distribution of SNPs in 16 *P. obconica* subsp. *obconica* accessions. (**A**) Distribution of SNPs across protein-coding genes; (**B**) Distribution of SNPs across intergenetic regions; (**C**) Number of SNPs shared by *P. obconica* subsp. *obconica*; (**D**) Distribution of SNPs shared by *P. obconica* subsp. *obconica*.

The clean data of 16 accessions were mapped onto the *Primula obconica* subsp. *obconica* (MK344754) chloroplast genome reference. A total of 1915 high-quality SNPs and 346 InDels were detected (Table S5). ZP2 harbored the largest number of genomic variants (1002), while the YN population (YN1 and YN2) possessed the fewest number of SNPs (335). The LSC region had a relatively higher number of SNPs compared with other regions. In the protein-coding gene region, there were 15 protein-coding genes which had an abundance of SNPs (*matK*, *atpF*, *rpoC2*, *rpoC1*, *rpoB*, *psaA*, *ycf3*, *clpP*, *rpl16*, *ndhF*, *ccsA*, *ndhD*, *ndhA*, *ndhH*, and *ycf1*). Moreover, *ycf1* contained the most abundant number of SNPs (133) (Figure 2A). In the intergenetic region, *matK-rps16*, *rps16-psbK*, *psbI-atpA*, *psbM-psbD*, *rps4-ndhJ*, *ndhC-atpE*, *rbcL-psaI*, *psbE-petL*, and *ndhF-rpl32* had more sequence variations, and the *psbM-psbD* intergenic spacers had the most abundant number of SNPs (Figure 2B). The results of SnpEff showed the total count of effects at 21371, and the plastome had an SNP density of 79 per bp. The ratio of non-synonymous to synonymous mutations was 1.1667. Most SNPs were detected in downstream and upstream gene regions (45.549% and 41.910%) (Table S6). One hundred and thirty SNPs were shared among all accessions. The Yunnan and Sichuan populations (YN, SCD, SCB) shared more variations in their genomic sequences than other accessions. The HN population possessed no species-specific SNPs, and the YN population possessed the most species-specific SNPs (Figure 2C). Most of the shared SNPs were detected in the *ycf1* gene (9), but when compared with the overall SNPs detected in the *ycf1* gene (133), it seems that the *ycf1* gene had more variations at the intraspecific level (Figure 2D).

### 3.3. Simple Sequence Repeat Analysis

A total of 401 SSRs were identified, including 317 mononucleotide, 31 dinucleotide, 39 tetranucleotide, and 14 pentanucleotide repeats (Figure S1A). Two cultivated species (ZP1 and ZP2) possessed the highest number of SSRs (51 and 49). Moreover, ZP1 and ZP2 possessed the greatest number of mononucleotide repeats when compared with other accessions. Mononucleotide repeat units (A/T) were the most abundant. Only one mononucleotide repeat unit (C), and one pentanucleotide repeat unit (TATAA) were highlighted in the cultivated accession of ZP1. Mononucleotide repeats were detected in ZP1, ZP2, HND1, and HNL1, respectively. Moreover, tetranucleotide repeat units (AAAT) only appeared in HBE1. However, no SSR motifs for tri- and hexanucleotide repeats were identified (Figure S1B). We found 15 SSRs that were shared in the nine accessions, twelve of which showed length variations between accessions (Table S7).

### 3.4. IR Expansion and Contraction

**Figure 3:**
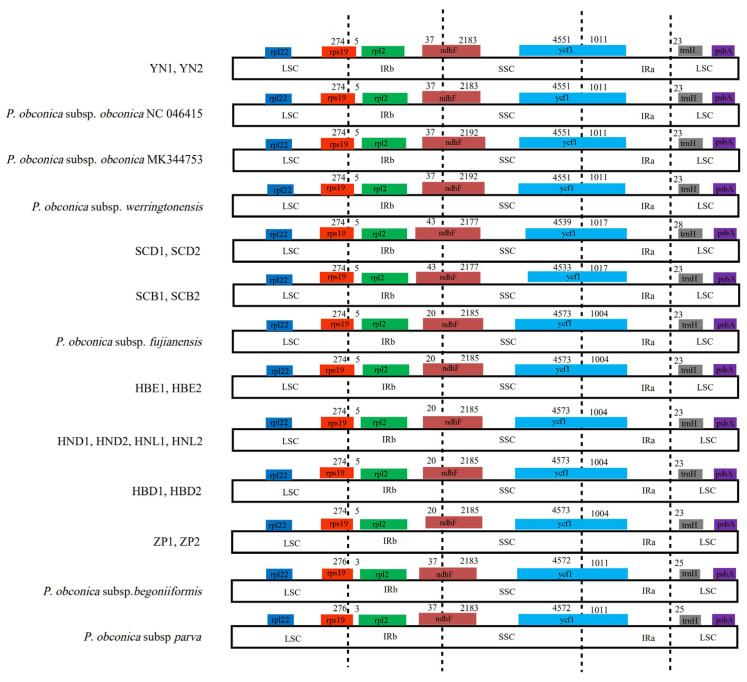
Comparisons of LSC, SSC, and IRs junctions among the *P. obconica* chloroplast genomes. Color codes: black dotted line, IR/LSC and IR/SSC boundary.

Studies on the expansion and contraction variation in junction regions between separate species are common but rarely focused on the intraspecific level. Although differently geographically distributed accessions of *Primula* subsp. *obconica* exhibited the same pattern in content and the number of genes in the IR/LSC and IR/SSC boundary regions, some differences still existed (Figure 3), such as the *ndhF* gene spanning the IRb/SSC border and the length ranging from 2177 to 2192 bp. The *ycf1* gene spanned the SSC/IRa border, from 4533 bp to 4573 bp in the SSC region, and from 1004 bp to 1017 bp in the IRa region. The *rps19* gene was located in the LSC region and extended 5 bp in the IRb region, except for *P. obconica* subsp. *parva* and *P. obconica* subsp. *begoniiformis* with 3 bp extended.

### 3.5. Comparative Analysis of the Chloroplast Genomes

**Figure 4:**
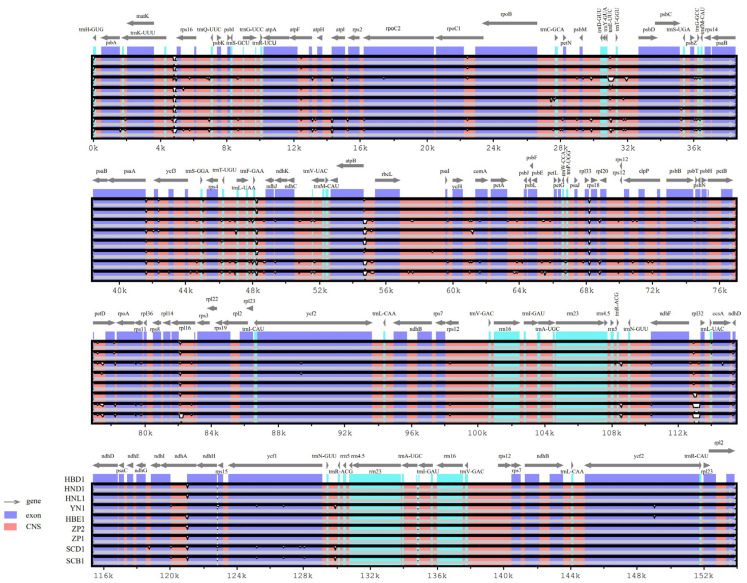
Comparison of *P. obconica* subsp. *obconica* plastome genomes using mVISTA, using HBD1 as a reference. Blue represents coding regions, Green represents RNA regions, and red represents non-coding regions.

The sequence divergences among the nine plastome genomes of *Primula obconica* subsp. *obconica* were compared, with HBD1 used as a reference (Figure 4). In general, as with the majority of angiosperm plastomes, the coding regions were more conserved than the non-coding regions. The LSC had a higher genetic variability than the LSC and IR regions. In the protein-coding regions, *atpF*, *petD*, *rpl16*, *ndhA*, and *ycf1* were the most variable genes. In the intergenic regions, *trnK-UUU-rps16*, *trnE-UUC-trnT-GGU*, *trnT-GGU-psbD*, *psaA-ycf3*, *trnF-GAA-ndhJ*, *atpB-rbcL*, *rpl33-rps18*, and *ndhF-rpl32* had a higher divergence. Furthermore, five subspecies of *P. obconica* were also compared, with HBD1 as the reference (Figure S2). Most variations were found in the intergenic regions, such as *rps16-trnQ-UUC*, *atpF-atpH*, *psbM-trnD-GUU*, *trnE-UUC-trnT-GGU*, *psaA-ycf3*, *ycf3-trnS-GGA*, *atpB-rbcL*, *rbcL-psaI*, *ycf4-cemA*, *psbE-petL*, and *rpl33-rps8*. The divergent regions of the protein-coding genes of these subspecies were consistent with *P. obconica* subsp. *obconica*, indicating that the protein-coding regions were more conserved than the intergenic regions.

### 3.6. Population Structure Analysis

**Figure 5:**
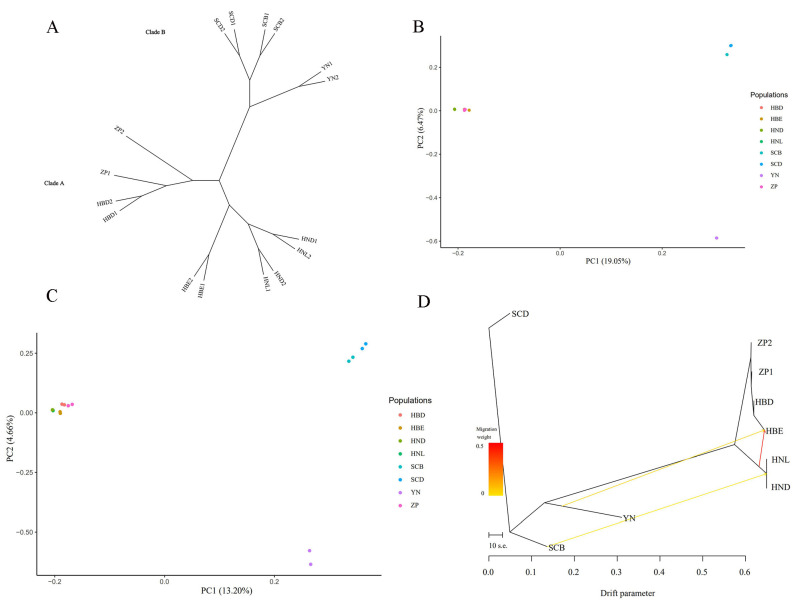
The genetic structure of 16 *P. obconica* subsp. *obconica*. (**A**) ML phylogenetic tree constructed using SNPs; (**B**) Principal component analysis based on SNPs; (**C**) Principal component analysis based on InDels; (**D**) TreeMix analysis and gene flow detected in *P. obconica* subsp. *obconica.* with SCD as the outgroup population.

In total, 1361 SNPs and 321 InDels were used to investigate genomic evolution among the 16 *Primula obconica* subsp. *obconica* after filtration. We constructed an ML phylogenetic tree based on SNP data (Figure 5A). Two major branches are shown in the phylogenetic trees. The Sichuan (SCB and SCD) and Yunnan populations (YN) were gathered in one clade, implying a close relationship between the two populations. The Hubei populations (HBE and HBD), the cultivated accessions (ZP1 and ZP2), and the Hunan populations (HND and HNL) showed closely-related relationships. A PCA analysis based on the SNPs and InDels was also performed to investigate the population genetic structure in 16 *P. obconica* subsp. *obconica* accessions (Figure 5B,C). The 16 *P. obconica* subsp. *obconica* accessions could be divided into two groups, which were consistent with topology A of the phylogenetic tree. To understand the history of the divergence, TreeMix was applied to investigate the migration events within the *P. obconica* subsp. *obconica* accessions, with the SCD population as the outgroup taxa (Figure 5D). The TreeMix result showed a relatively high gene flow that appeared in the HBE and Hunan populations (HNL and HND). A weak gene flow from SCB to the Hunan populations and YN to HBE was observed. Furthermore, we did not observe gene flow between the cultivated accessions, and the SCD and HBD populations. 

### 3.7. Phylogenetic Analyses

**Figure 6:**
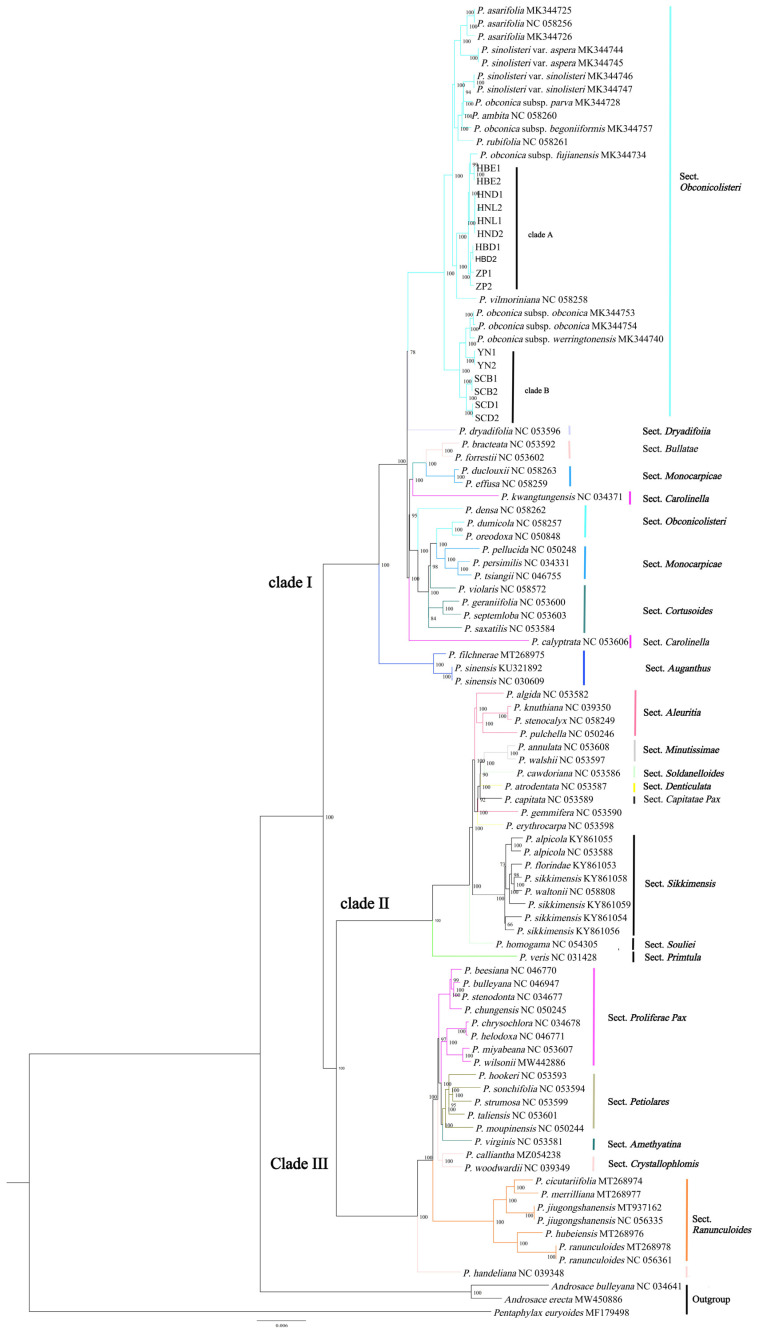
Molecular phylogenetic tree of *Primula* using maximum likelihood methods based on 77 shared protein-coding genes.

An ML tree was constructed using 77 protein-coding genes that were shared by 100 accessions (Figure 6). The vast majority of the nodes in the phylogenetic trees received more than 90% bootstrap support values. Three major branches are shown in the phylogenetic trees, both with identical tree topologies. 

Cluster I contained seven sections, including Sect. *Obconicolisteri*, Sect. *Dryadifoiia*, Sect. *Bullatae*, Sect. *Monocarpicae*, Sect. *Carolinella*, Sect. *Cortusoides*, and Sect. *Auganthus*. The Sect. *Obconicolisteri* was a sister to the clade Sect. *Dryadifoiia* with strong support (100%), but three species of Sect. *Obconicolisteri* (*P. densa*, *P. dumicola*, *P. oreodoxa*) were more closely related to Sect. *Monocarpicae* (*P. pellucida*, *P. persimilis*, *P. tsiangii*) and Sect. *Cortusoides*. *P. obconica* subsp. *obconica* was not a monophyletic species in our results, it was divided into two distinct related clades. Two subspecies (*P. obconica* subsp. *parva*, *P. obconica* subsp. *begoniiformis*) of *P. obconica* were clustered into a separate branch. The Sect. *Bullatae* was clustered to the rest of the Sect. *Monocarpicae*. *P. calyptrata* belonged to Sect. *Carolinella*, forming a separate branch. The result supported the hypothesis that Sect. *Auganthus* and Sect. *Bullatae* comprise a monophyletic section. 

Cluster II contained eight sections, including Sect. *Aleuritia*, Sect. *Minutissimae*, Sect. *Soldanelloides*, Sect. *Denticulata*, Sect. *Capitatae Pax*, Sect. *Sikkimensis*, Sect. *Souliei*, and Sect. *Primula*. Four of five species of Sect. *Aleuritia* clustered to one clade, but the placement of *P. gemmifera* (taxonomically ascribed to Sect. *Aleuritia*) in the phylogenetic tree resulted in the non-monophyly of Sect. *Aleuritia*. The Sect. *Minutissimae* and Sect. *Soldanelloides* clustered to a sister group. Three sections (Sect. *Denticulata*, Sect. *Capitatae Pax*, Sect. *Aleuritia*) were mixed in the phylogenetic tree. Our result only supported Sect. *Sikkimensis* and Sect. *Minutissimae* forming a monophyletic group in cluster II. 

The rest of the sections (Sect. *Proliferae Pax*, Sect. *Petiolares*, Sect. *Amethyatina*, Sect. *Crystallophlomis*, Sect. *Ranunculoides*) formed cluster III, in which Sect. *Proliferae Pax* and Sect. *Crystallophlomis* did not form a monophyletic section. Four species (*P. chrysochlora*, *P. helodoxa*, *P. miyabeana*, *P. wilsonii*) in Sect. *Proliferae Pax* formed a clade sister to Sect. *Amethyatina*. One species is of special interest in cluster III: *P. handeliana* is ascribed to Sect. *Crystallophlomis*, but it formed a distinct clade with Sect. *Crystallophlomis* and was more closely related to Sect. *Ranunculoides*. The monophyly of Sect. *Ranunculoides* is strongly supported in this study.

Several sections were represented by only one species, including Sect. *Dryadifoiia*, Sect. *Soldanelloides*, Sect. *Capitatae Pax*, Sect. *Souliei*, Sect. *Primtula*, and *Sect. Amethyatina*. Sect. *Dryadifoiia* formed a sister clade to Sect. *Obconicolisteri*. Sect. *Capitatae Pax* clustered together among the Sect. *Aleuritia* and Sect. *Denticulata*. Sect. *Primtula* formed a separate branch. Our result was insufficient to infer whether these sections are monophyletic.

## 4. Discussion

Plastome variations within species have rarely been reported. The length of plastomes, the gene number, and the content within species were conserved. The plastomes of *P. obconica* all contain 128 genes, including 83 protein-coding genes, eight ribosomal RNAs, and 37 transfer RNAs. Prior research has indicated that the length variation in the whole plastome is mainly affected by the difference of LSC region [7], but our results showed that both the LSC and SSC regions play an important role in plastome size variation within species. The LSC region of HBE1 was 309 bp less in size than HBL2 and HBD1. The SSC region among HBL2, HBD1, and SCD1 varied by 396 bp in length, and the IR regions were more conserved, with only 48-bp length variations at the intraspecific level. The length varied between different accessions of *P. obconica* subsp. *obconica*, which may be affected by geographical isolation.

Although plastomes have the properties of uniparental inheritance and a low frequency of recombination, these features have also prompted plastomes to maintain highly conserved features; however, mutations at the intraspecific level have still occurred, such as in *Eragrostis tef* (12 InDels and 19 SNPs) [43], *Selaginella tamariscina* (1641 InDels and 1213 SNPs) [44], and five *Goodyera schlechtendaliana* (414-2133 InDels and 200-844 SNPs) [45]. A total of 1915 high-quality SNPs and 346 InDels were detected in 16 accessions of *P. obconica* subsp. *obconica*. The cultivated accession ZP2 harbored the largest number of SNPs (1002), and wild accessions of YN (YN1 and YN2) had the fewest number of SNPs (335). In contrast, the genomic variants detected in wild rubber trees (193) were much larger than those detected in cultivated (91) accessions [46]. Many of the morphological characteristics shown in the cultivated accessions were different from the wild populations; for example, the leaves and flowers were larger than those of the wild accessions. Moreover, the inferred ML tree displayed more distant relationships between ZP and *P. obconica* subsp. *obconica* (MK344754) than other accessions. Among the protein-coding genes, 15 genes had a relatively higher number of SNPs (*matK*, *atpF*, *rpoC2*, *rpoC1*, *rpoB*, *psaA*, *ycf3*, *clpP*, *rpl16*, *ndhF*, *ccsA*, *ndhD*, *ndhA*, *ndhH*, and *ycf1*). Moreover, SNPs were especially abundant in the *ycf1* gene (133), which was also reported in *Ricinus communis* [7]. The *matK*, *psbA*, *ndhF*, and *ycf1* genes were used as cpDNA barcodes to identify closely related species [47,48,49,50]. Moreover, the *ycf1* gene was considered as the core barcode of land plants [50]. These cpDNA barcodes not only greatly varied between the interspecies of the genus *Primula* but were also varied among the different varieties of *P. obconica* [51]. In total, 1915 SNPs were detected in 16 plastome genomes of *P. obconica* subsp. *obconica*, but only 131 SNPs were common, which showed higher variation at the intraspecific level of *P. obconica* subsp. *obconica*. These samples were collected from different geographic locations, and climatic variation may be the main driver triggering genomic variations [52]. The SnpEff annotation results showed that most of the SNPs were distributed in the downstream and upstream regions (45.549% and 41.910%), which was consistent with the Triticum-Aegilops complex plastome genomes [53].

Due to their high abundance and variability, especially of the repeat and motif structures of SSRs in the cp genome, the SSRs have been used for population genetics and evolution studies [54,55]. Seven individuals from different geographic locations and two cultivated accessions were analyzed. A total of 401 SSRs were detected, and two cultivated accessions had the highest abundance of SSRs, which was consistent with cultivated rubber [46]. Our results are consistent with previous research, which described that mononucleotide repeats representing the most common repeat type of SSRs and chloroplasts generally consisted of short polyA/T repeats [56,57]. Furthermore, 15 SSRs shared in the nine accessions were detected, and 12 of these had length variations between those accessions. These variations may be helpful for population genetic analysis in *P. obconica* subsp. *obconica*.

Similar to other angiosperm studies, protein-coding sequences were more conserved than intergenic regions, and the LSC regions were the most divergent [58,59]. In the protein-coding region, five genes showed increased variations, including *atpF*, *petD*, *rpl16*, *ndhA*, and *ycf1*. These five genes were in both the 16 *P. obconica* subspecies and four subspecies. In the intergenic regions, the four subspecies showed more divergence in *rps16-trnQ-UUC*, *atpF-atpH*, *psbM-trnD-GUU*, *ycf3-trnS-GGA*, *rbcL-psaI*, *ycf4-cemA*, and *psbE-petL*. These variation regions were different from those detected within *P. obconica* subsp. *obconica*, indicating the divergence between the four subspecies and *P. obconica* subsp. *obconica*.

The expansion and contraction between IR and the single-copy (SC) boundary regions are considered the main causes of the cp genome variation [60]. There has been considerable research indicating that genes in the IR and single-copy boundary regions vary in different taxa [61,62], such as in cultivated and wild *Hevea brasiliensis*, in which there are variations in gene number and the length of *ycf1* spanning the boundary of the SSC region [46]. In our study, the junctions between the IR and single-copy boundary regions were highly conserved between the wild and cultivated *P. obconica* subsp. *obconica*. There were apparent variations in some genes spanning the boundary of the SC/IR region. These variations are likely associated with phylogenetic signals, for example, the *ndhF* spanning the boundary of the IRb/SSC region was consistent with the clade of the phylogenetic tree in *P. obconica* subsp. *obconica*.

Southwest China is considered the biodiversity center of *Primula* and the place of origin of other land plants [63,64]. Migration events between Southwest China and East China may occur along several mountain ranges, such as the Qin-Daba Mountains and Dalou Mountains [65]. Considerable research efforts have demonstrated that *P. obconica* originated from the Yunnan and Sichuan provinces [66,67]. Two major branches of *P. obconica* were shown in phylogenetic trees and PCA analysis, which were based on data regarding SNPs, InDels, and 77 shared protein-coding genes. Our results were consistent with the finding that *P. obconica* was split into the Sichuan-Central group and Yunnan-Eastern group [67]. There are a series of high mountains that may be a geographical barrier between Yunnan and Sichuan, which may have triggered intraspecies genetic divergence between the Yunnan lineages and Sichuan lineages [68]. Our phylogenetic tree showed that *P. obconica* subsp. *obconica* was separated into two groups, consistent with the result of our SSR analysis [69]. Non-monophyletic species make it difficult to infer phylogenetic relationships [70]. Whether monophyly can be used to define species is also controversial and non-monophyletic species have also been considered as “wrong” taxonomy [71]. However, non-monophyletic species occur in many taxa of the family Primulaceae, such as *Dodecatheon dentatum*, *Dodecatheon jeffreyi*, *Primula veris*, and *Primula vulgaris* [72,73]. The explanations for non-monophyletic species are hybridization, ancestral polymorphism, or both processes [72]. Moreover, geographical isolation and environmental heterogeneity each play a crucial role in species diversification [74]. Based on the insufficiency of our data, it is not possible to reliably infer the reasons behind the non-monophyletic occurrence of *P. obconica* subsp. *obconica*. 

Many factors can affect genetic diversity within species, such as geographic distribution, breeding system, and isolation by gene flow [75]. Gene flow plays an important role in the speciation and adaptation of land plants [76]. The extent of gene flow between species could be confused with the interspecific boundaries and can bring difficulties to species delimitation [77]. In contrast, frequent gene flow has occurred within species, which could reduce intraspecific divergence, trends which are helpful for species delimitation [78]. High levels of genetic variation are common in the genus *Primula*, such as *P. sikkimensis* (Shannon’s index *H*_SP_: 0.5576, expected heterozygosity Hj: 0.4032) [75], and *P. interjacens* (*H*_SP_: 0.4618) [79]. According to previous research, there is a lower genetic diversity within populations than between populations of *P. obconica*. Prior research has implied that the genetic differentiation in *P. obconica* was mainly caused by geographical barriers and seed dispersal mechanisms (dispersed by gravity, ants, or rodents) [67,80]. In this study, a relatively high gene flow had appeared from the Hunan populations (HNL and HND) to HBE. The two populations had closely related relationships and geographical distances. Low-frequency gene flow was detected in clades A and B, which confirmed the results of the geographical barriers and seed dispersal mechanisms, exacerbating the process of divergence in *P. obconica* subsp. *obconica*. 

*P. obconica* subsp. *werringtonensis* is distributed in the west of Sichuan, with a margin of usually sinuate-lobulate that is distinguished from *P. obconica* subsp. *obconica*. *P. obconica* subsp. *begoniiformis* possesses slender petioles, a leaf blade of ovate-rotund to suborbicular shape, and a margin of crenate-lobulate that differs from *P. obconica* subsp. *obconica*. *P. obconica* subsp. *parva* and *P. obconica* subsp. *fujianensis* all have the characteristic of scapes that are shorter than leaves. The four subspecies all have very small populations and are restricted to the Yunnan, Sichuan, and Fujian provinces of the Chinese mainland. A previous study inferred that *P. obconica* subsp. *fujianensis* originated from *P. obconica* subsp. *obconica*, and the east of Yunnan populations originated from the Sichuan population. Moreover, the variation in genetics between the four subspecies and *P. obconica* subsp. *obconica* was not obvious [25]. In our phylogenetic tree result, only *P. obconica* subsp. *fujianensis* and *P. obconica* subsp. *werringtonensis* were nested within *P. obconica* subsp. *obconica*, suggesting the two subspecies had genetic variation overlapping the *P. obconica* subsp. *obconica* populations. Additionally, *P*. *obconica* subsp. *parva* and *P. sinolisteri* subsp. *sinolisteri* were clustered in a sister clade, while *P. obconica* subsp. *begoniiformis* and *P. ambita* were closely related. The two subspecies have highly recognizable morphological characteristics, isolated geographical distribution areas, and a distinct phylogenetic relationship compared with *P. obconica* subsp. *obconica*. We propose the elevation of the two subspecies of *P. obconica* to separate species, *P. begoniiformis* and *P. parva*.

Our phylogenetic tree showed multiple non-monophyletic sections of *Primula*, such as Sect. *Obconicolisteri*, Sect. *Monocarpicae*, Sect. *Cortusoides*, Sect. *Carolinella*, Sect. *Aleuritia*, Sect. *Denticulata*, and Sect. *Crystallophlomis*. Three possible explanations may aid in the interpretation of these phenomena. Firstly, genetic drift, ancestral polymorphism, polyploidization, and recent introgression lead to sister species becoming reciprocally monophyletic. Gene exchange among interspecies may lead to the combination of genetic components of different ancestors, in which case, phylogenetic analysis between hybrids will be more challenging. Previous research has demonstrated that natural hybridization is common and has been detected between interspecies in *Primula* [21,81,82]. The main pollinators of *Primula* species were bees and butterflies, there were 12 families and 22 species of flower-visiting insects of *Primula lithophila* [83]. Multiple pollinators have increased the opportunity of *Primula* outcrossing. Polyploidization has been detected in *Primula*, suggesting there was different ploidy in or between sections [84]. Furthermore, polyploidization has also caused non-monophyletic species in the genus *Aconitum* [85,86,87]. Phylogenetic genetic analysis revealed tetraploid *P. egaliksensis* (taxonomically ascribed to Sect. *Armerina*) were clustered to Sect. *Aleuritia* [88]. Polyploidization and hybridization between other natural sections will need extensive analyses to reveal whether these events are responsible for the non-monophyly of the phylogeny. Secondly, it may be a wrong taxonomy. Morphological classification possesses strong subjectivity based on taxonomists. Besides, phenotypes are susceptible to environmental effects, such as convergent evolution, leading to two distant species evolving similar morphological characteristics. The calyptrate capsule that occurred in the genera *Pomatosace*, *Anagallis*, *Soldanella*, and *Bryocarpum* in Primulaceae has been attributed to convergent evolution [89]. Furthermore, *P. secundiflora* has long been treated as a member of Sect. *Sikkimensis* based on the campanulate corolla, but phylogenetic analysis suggested it should be placed in Sect. *Proliferae Pax* [90]. Recently, *P. subansirica* (taxonomically ascribed to Sect. *Sikkimensis*) was found to belong to Gesneriaceae [91]. Thirdly, chloroplast capture through hybridization may occur in these sections. Chloroplast capture is common in plants and also has been detected in the genus *Primula* [92,93,94]. 

Richard (2003) recognized seven subgenera and thirty-eight sections in *Primula* worldwide [23]. Our phylogenetic tree was largely inconsistent with morphology-based taxonomy. Twenty-one sections were divided into three main clades in our results. Clade I contained major sections of subgen. *Auganthus*; only one section belonging to subgen. *Carolinella* was nested within subgen. *Auganthus*. Our phylogenetic analysis implied neither subgen. *Auganthus* nor subgen. *Carolinella* are monophyletic taxa, consistent with previous analyses [10,95,96]. The subgen. *Carolinella* with its calyptrate capsule can be distinguished from other subgenera. Previous research has implied that the calyptrate capsule might have evolved multiple times in *Primula* [96]. We agree with this view because *P. calyptrata* and *P. kwangtungensis* were separated from each other and distantly related. Previous studies have also been performed on Sect. *Obconicolisteri*. These results were inconsistent with our study, due to insufficient samples and not being combined with other sections in previous phylogenetic analyses [10,16,69]. Our results demonstrated that Sect. *Obconicolisteri* is not a monophyletic group. Three species of this section were clustered with Sect. *Monocarpicae* and Sect. *Cortusoides*. To make Sect. *Obconicolisteri* monophyletic, these three species should be excluded. *P. dumicola* and *P. oreodoxa* were treated as members of Sect. *Obconicolisteri*, but appear closer to Sect. *Monocarpicae*. Regarding the two species with calyx broadly campanulate and *P. dumicola* with calyx margin entire, these characteristics are more similar to Sect. *Monocarpicae*. Besides, *P. calyptrata* (taxonomically ascribed to Sect. *Carolinella*) and *P. handeliana* (taxonomically ascribed to Sect. *Crystallophlomis*) possess a distant phylogenetic relationship with their natural morphology sections. Phylogenetic analyses based on protein-coding genes were also performed by Ren (2018), implying that *P. calliantha* and *P. woodwardii* did not have the closest relationship [97]. Molecular data and morphological cluster analysis based on population samples will be needed to infer whether it is a wrong taxonomy, or can be explained by chloroplast genome capture, introgression, or other evolution events.

Clade II included subgen. *Primula* (Sect. *Primula*) and subgen. *Aleuritia*. The subgenus *Aleuritia* is a large group in *Primula* with 19 sections. Our results were largely congruent with previous research that subgen. *Aleuritia* is paraphyletic [98,99]. *P. gemmifera* (taxonomically ascribed to Sect. *Aleuritia*) is endemic to China, mainly distributed in southern Gansu, western Sichuan, and northeastern Tibet. In our results, *P. gemmifera* is grouped with Sect. *Capitatae Pax* and Sect. *Denticulata* and form a well-supported clade. The non-monophyly of Sect. *Aleuritia* is consistent with the conclusion made by Ren (2015) [100]. However, the monophyly of sections in *Aleuritia* is also supported by other research [88,101], though these results were due to insufficient samples and a lack of comparative analysis with relevant groups. Furthermore, the monophyly of Sect. *Sikkimensis* is well supported in our result. 

Clade III contained subgen. *Pinnatae* (Sect. *Ranunculoides*), subgen. *Aleuritia*, and subgen. *Auriculastrum* (Sect. *Amethyatina*). The results strongly supported the monophyly of Sect. *Ranunculoides* and Sect. *Petiolares*. Four species (*P. chrysochlora*, *P. helodoxa*, *P. miyabeana*, *P. wilsonii*) were clustered to Sect. *Petiolares* and Sect. *Amethyatina*. The Sect. *Proliferae Pax* was paraphyletic. The result differed from cpDNA and ITS analyses, owing to the absence of species of Sect. *Petiolares* and Sect. *Amethyatina* in previous research. Hu and Kelso (1996) renamed Sect. *Pinnatae* as a new section, Sect. *Ranunculoides*, and transferred *P. filchnerae* into Sect. *Auganthus*. Subsequently, this treatment was authenticated by molecular phylogenetic analysis [102]. Our phylogenetic tree also supported the placement of *P. filchnerae* in Sect. *Auganthus*. Furthermore, Sect. *Auganthus* and Sect. *Ranunculoides* were two monophyletic and distantly related sections.

## 5. Conclusions

Although chloroplast genomes have the properties of uniparental inheritance and low frequency of recombination, these features have prompted plastomes to possess highly conserved features. However, variations at the intraspecies level have still occurred. Sixteen plastome genomes of *P. obconica* subsp. *obconica* were identified to have different lengths, which were mainly caused by variations in the LSC and SSC regions. Highly genome-wide variations were detected. Most SNPs were detected in downstream and upstream gene regions (45.549% and 41.91%). Multiple sequence divergent regions and SSRs found in this study would provide useful information for population genetic analyses in *P. obconica*. The phylogenetic tree showed that *P.* subsp. *obconica* can be split into two independent clades. The taxonomy of the *P. obconica* complex was redefined in this text. We elevated two subspecies of *P. obconica* to separate species. Our phylogenetic tree was largely inconsistent with the previous morphology-based taxonomy. Twenty-one sections of *Primula* were mainly divided into three clades. The monophyly of Sect. *Auganthus*, Sect. *Minutissimae*, Sect. *Sikkimensis*, Sect. *Petiolares*, and Sect. *Ranunculoides* were well supported in the phylogenetic tree. The Sect. *Obconicolisteri*, Sect. *Monocarpicae*, Sect. *Carolinella*, Sect. *Cortusoides*, Sect. *Aleuritia*, Sect. *Denticulata*, Sect. *Proliferae Pax*, and Sect. *Crystallophlomis* were not a monophyletic group. Several sections were represented by only one species, the phylogenetic relationships of these sections will need a larger number of samples in future studies. 

## Data Availability

The chloroplast genome sequences have been deposited in GenBank under the accession numbers: OK040681, OK040682, OK040683, OK040684, OK040685, OK040686, OK040687, OK040688, OK040689, OK040690, OK040691, OK040692, OK040693, OK040694, OK040695, OK040696. Row data are available at SRA, under the accession number: PRJNA789333.

## Supplementary Materials

The following supporting information can be downloaded at: https://www.mdpi.com/article/10.3390/genes13040567/s1, Figure S1: The presence of simple sequence repeats (SSRs) in the *P. obconica* subsp. *obconica* chloroplast genomes. (A) The number of repeats types; (B) Frequency of variations SSRs types, Figure S2: Comparison of 5 subspecies of *P. obconica* plastome genomes using mVISTA, Table S1: Details of accessions used in the study, Table S2: List of genes in the plastome genomes of *P. obconica* subsp. *obconica*, Table S3: The lengths of introns and exons for the splitting genes in *P. obconica* subsp. *obconica*, Table S4: SNPs identified in *P. obconica* subsp. *obconica* plastomes, Table S5: SNP annotation based on 16 plastome genomes of *P. obconica* subsp. *obconica* using SnpEff, Table S6: List of SSRs that are shared by *P. obconica* subsp. *obconica*, Table S7: List the SSRs that shared by *P. obconica* subsp. *obconica*.

## References

[1] Shang H., Liu B., Kang M., Yan Y. (2019). One or more species? GBS sequencing and morphological traits evidence reveal species diversification of *Sphaeropteris brunoniana* in China. Biodivers. Sci..

[2] Murphy D.J., Brown G.K., Miller J.T., Ladiges P.Y. (2010). Molecular phylogeny of *Acacia Mill*. (Mimosoideae: Leguminosae): Evidence for major clades and informal classification. Taxon.

[3] Wang Y., Wang S., Liu Y., Yuan Q., Sun J., Guo L. (2021). Chloroplast genome variation and phylogenetic relationships of *Atractylodes* species. BMC Genom..

[4] Ji Y., Yang J., Landis J.B., Wang S., Yang Z., Zhang Y. (2021). Deciphering the Taxonomic Delimitation of *Ottelia acuminata* (Hydrocharitaceae) Using Complete Plastomes as Super-Barcodes. Front. Plant Sci..

[5] Boutte J., Maillet L., Chaussepied T., Letort S., Aury J.M., Belser C., Boideau F., Brunet A., Coriton O., Deniot G. (2020). Genome Size Variation and Comparative Genomics Reveal Intraspecific Diversity in *Brassica rapa*. Front. Plant Sci..

[6] Park J., Xi H., Kim Y. (2020). The Complete Chloroplast Genome of *Arabidopsis thaliana* Isolated in Korea (Brassicaceae): An Investigation of Intraspecific Variations of the Chloroplast Genome of Korean *A. thaliana*. Int. J. Genom..

[7] Muraguri S., Xu W., Chapman M., Muchugi A., Oluwaniyi A., Oyebanji O., Liu A. (2020). Intraspecific variation within Castor bean (*Ricinus communis* L.) based on chloroplast genomes. Ind. Crops Prod..

[8] Smith D.R. (2015). Mutation rates in plastid genomes: They are lower than you might think. Genome Biol. Evol..

[9] Hu C., Kelso S. (1996). Flora of China.

[10] Mast A.R., Kelso S., Richards A.J., Lang D.J., Feller D.M.S., Conti E., Journal I., Mast A.R., Kelso S., Richards A.J. (2001). Phylogenetic relationships in *Primula* L. and related genera (Primulaceae) based on noncoding chloroplast DNA. Int. J. Plant Sci.

[11] De Vos J.M., Hughes C.E., Schneeweiss G.M., Moore B.R., Conti E. (2014). Heterostyly accelerates diversification via reduced extinction in primroses. Proc. R. Soc. B Biol. Sci..

[12] Nuraliev M.S., Kuznetsov A.N., Kuznetsova S.P., Hu C.M. (2020). *Primula gracilituba* (Primulaceae), a new species from northern Vietnam. Nord. J. Bot..

[13] Ma X.D., Wang W.G., Shi J.P., Shen J.Y. (2021). *Primula longistyla* (Primulaceae), a new species from Yunnan, China. Nord. J. Bot..

[14] Ju W.B., Huang Q., Sun Z.Y., Huang W.J., Li H.C., Gao X.F. (2018). *Primula luteoflora* (Primulaceae), a new species from Sichuan, China. Phytotaxa.

[15] Zhang L.B., Comes H.P., Kadereit J.W. (2004). The temporal course of quaternary diversification in the European high mountain endemic *Primula* sect. *Auricula* (Primulaceae). Int. J. Plant Sci..

[16] Yan H.F., Liu Y.J., Xie X.F., Zhang C.Y., Hu C.M., Hao G., Ge X.J. (2015). DNA barcoding evaluation and its taxonomic implications in the species-rich genus *Primula* L. in China. PLoS ONE.

[17] Li J., Li M., Zhu W., Zhang X. (2020). The reproductive characteristics of *Primula obconica*. J. Hunan Agric. Univ..

[18] Yuan S., Barrett S.C.H., Duan T., Qian X., Shi M., Zhang D. (2017). Ecological correlates and genetic consequences of evolutionary transitions from distyly to homostyly. Ann. Bot..

[19] Chen M., You Y., Zhang X. (2010). Advances in the research of heterostyly. Acta Prataculturae Sin..

[20] Tendal K., Ørgaard M., Larsen B., Pedersen C. (2018). Recurrent hybridisation events between *Primula vulgaris*, *P. veris* and *P. elatior* (Primulaceae, Ericales) challenge the species boundaries: Using molecular markers to re-evaluate morphological identifications. Nord. J. Bot..

[21] Cianchi R., Arduino P., Mosco M.C., Bullini L. (2015). Evidence of hybrid speciation in the North American primroses *Primula suffrutescens P. parryi P. rusbyi* and *P. angustifolia* (Primulaceae). Plant Biosyst..

[22] He G.-S., Hu C.-M. (2002). A new subspecies of *Primula obconica* Hance from Eastern China. Acta Phytotaxon. Sin..

[23] Richards J. (2002). Primula (New Edition).

[24] Smith W., Forrest G. (1928). The sections of the genus *Primula*. Notes from the Royal Botanic Garden.

[25] Yan H., Wang X.L., Hu Q., Hao G. (2005). Phylogeography of *Primula obconica* Hance (Primulaceae). J. Trop. Subtrop. Bot..

[26] Nan P., Peng S., Zhang Y., Zhong Y. (2002). Composition of volatile oil of *Primula obconica* in central China. Nat. Prod. Lett..

[27] Mowad C.M. (1998). Routine testing for *Primula obconica*: Is it useful in the United States?. Am. J. Contact Dermat..

[28] Yan H.F., Ge X.J., Hu C.M., Hao G. (2010). Isolation and characterization of microsatellite loci for the ornamental plant *Primula obconica* Hance (Primulaceae). HortScience.

[29] Jin J.J., Yu W.B., Yang J.B., Song Y., DePamphilis C.W., Yi T.S., Li D.Z. (2020). GetOrganelle: A fast and versatile toolkit for accurate de novo assembly of organelle genomes. Genome Biol..

[30] Wick R.R., Schultz M.B., Zobel J., Holt K.E. (2015). Bandage: Interactive visualization of de novo genome assemblies. Bioinformatics.

[31] Shi L., Chen H., Jiang M., Wang L., Wu X., Huang L., Liu C. (2019). CPGAVAS2, an integrated plastome sequence annotator and analyzer. Nucleic Acids Res..

[32] Greiner S., Lehwark P., Bock R. (2019). OrganellarGenomeDRAW (OGDRAW) version 1.3.1: Expanded toolkit for the graphical visualization of organellar genomes. Nucleic Acids Res..

[33] Li H., Durbin R. (2009). Fast and accurate short read alignment with Burrows-Wheeler transform. Bioinformatics.

[34] Li H., Handsaker B., Wysoker A., Fennell T., Ruan J., Homer N., Marth G., Abecasis G., Durbin R. (2009). The Sequence Alignment/Map format and SAMtools. Bioinformatics.

[35] Depristo M.A., Banks E., Poplin R., Garimella K.V., Maguire J.R., Hartl C., Philippakis A.A., Del Angel G., Rivas M.A., Hanna M. (2011). A framework for variation discovery and genotyping using next-generation DNA sequencing data. Nat. Genet..

[36] Cingolani P., Platts A., Wang L.L., Coon M., Nguyen T., Wang L., Land S.J., Lu X., Ruden D.M. (2012). A program for annotating and predicting the effects of single nucleotide polymorphisms, SnpEff: SNPs in the genome of Drosophila melanogaster strain w1118; iso-2; iso-3. Fly.

[37] Beier S., Thiel T., Münch T., Scholz U., Mascher M. (2017). MISA-web: A web server for microsatellite prediction. Bioinformatics.

[38] Lischer H.E.L., Excoffier L. (2012). PGDSpider: An automated data conversion tool for connecting population genetics and genomics programs. Bioinformatics.

[39] Minh B.Q., Schmidt H.A., Chernomor O., Schrempf D., Woodhams M.D., Von Haeseler A., Lanfear R., Teeling E. (2020). IQ-TREE 2: New Models and Efficient Methods for Phylogenetic Inference in the Genomic Era. Mol. Biol. Evol..

[40] Purcell S., Neale B., Todd-Brown K., Thomas L., Ferreira M.A.R., Bender D., Maller J., Sklar P., De Bakker P.I.W., Daly M.J. (2007). PLINK: A tool set for whole-genome association and population-based linkage analyses. Am. J. Hum. Genet..

[41] Pickrell J.K., Pritchard J.K. (2012). Inference of Population Splits and Mixtures from Genome-Wide Allele Frequency Data. PLoS Genet..

[42] Zhang D., Gao F., Jakovlić I., Zou H., Zhang J., Li W.X., Wang G.T. (2020). PhyloSuite: An integrated and scalable desktop platform for streamlined molecular sequence data management and evolutionary phylogenetics studies. Mol. Ecol. Resour..

[43] Teshome G.E., Mekbib Y., Hu G., Li Z.Z., Chen J. (2020). Comparative analyses of 32 complete plastomes of Tef (*Eragrostis tef*) accessions from Ethiopia: Phylogenetic relationships and mutational hotspots. PeerJ.

[44] Park J., Kim Y., Lee G.H., Park C.H. (2020). The complete chloroplast genome of *Selaginella tamariscina* (Beauv.) Spring (Selaginellaceae) isolated in Korea. Mitochondrial DNA Part B Resour..

[45] Oh S.H., Suh H.J., Park J., Kim Y., Kim S. (2019). The complete chloroplast genome sequence of *Goodyera schlechtendaliana* in Korea (Orchidaceae). Mitochondrial DNA Part B Resour..

[46] Feng L.Y., Liu J., Gao C.W., Wu H.B., Li G.H., Gao L.Z. (2020). Higher Genomic Variation in Wild Than Cultivated Rubber Trees, Hevea brasiliensis, Revealed by Comparative Analyses of Chloroplast Genomes. Front. Ecol. Evol..

[47] Hilu K., Alice L., Liang H. (1999). Phylogeny of Poaceae inferred from matK sequences. Ann. Mo. Bot. Gard..

[48] Lehnebach C.A., Cano A., Monsalve C., McLenachan P., Hörandl E., Lockhart P. (2007). Phylogenetic relationships of the monotypic *Peruvian* genus *Laccopetalum* (Ranunculaceae). Plant Syst. Evol..

[49] Zhang L., Wang Y., Chen Q., Luo Y., Zhang Y., Tang H., Wang X. (2015). Phylogenetic Utility of Chinese *Rubus* (Rosaceae) Based on ndhF Sequence. Acta Hortic. Sin..

[50] Dong W., Xu C., Li C., Sun J., Zuo Y., Shi S., Cheng T., Guo J., Zhou S. (2015). ycf1, the most promising plastid DNA barcode of land plants. Sci. Rep..

[51] Xu W., Xia B., Li X. (2020). The complete chloroplast genome sequences of five pinnate-leaved Primula species and phylogenetic analyses. Sci. Rep..

[52] Cuevas A., Ravinet M., Sætre G.P., Eroukhmanoff F. (2021). Intraspecific genomic variation and local adaptation in a young hybrid species. Mol. Ecol..

[53] Fu Y.-B. (2021). Characterizing chloroplast genomes and inferring maternal divergence of the Triticum–Aegilops complex. Sci. Rep..

[54] Ellegren H. (2004). Microsatellites: Simple sequences with complex evolution. Nat. Rev. Genet..

[55] Taniguchi F., Kimura K., Saba T., Ogino A., Yamaguchi S., Tanaka J. (2014). Worldwide core collections of tea (*Camellia sinensis*) based on SSR markers. Tree Genet. Genomes.

[56] Liu H., Hu H., Zhang S., Jin J., Liang X., Huang B., Wang L. (2020). The complete chloroplast genome of the rare species *Epimedium tianmenshanensis* and comparative analysis with related species. Physiol. Mol. Biol. Plants.

[57] Wei F., Tang D., Wei K., Qin F., Li L., Lin Y., Zhu Y., Khan A., Kashif M.H., Miao J. (2020). The complete chloroplast genome sequence of the medicinal plant *Sophora tonkinensis*. Sci. Rep..

[58] Raman G., Park S.J. (2020). The complete chloroplast genome sequence of the *speirantha gardenii*: Comparative and adaptive evolutionary analysis. Agronomy.

[59] YANG C.H., LIU X., CUI Y.X., NIE L.P., LIN Y.L., WEI X.P., WANG Y., YAO H. (2020). Molecular structure and phylogenetic analyses of the complete chloroplast genomes of three original species of *Pyrrosiae Folium*. Chin. J. Nat. Med..

[60] Huang H., Shi C., Liu Y., Mao S.-Y., Gao L.-Z. (2014). Thirteen *Camellia* chloroplast genome sequences determined by high-throughput sequencing: Genome structure and phylogenetic relationships. BMC Evol. Biol..

[61] Kong B.L.H., Park H.S., Lau T.W.D., Lin Z., Yang T.J., Shaw P.C. (2021). Comparative analysis and phylogenetic investigation of Hong Kong *Ilex* chloroplast genomes. Sci. Rep..

[62] Zheng G., Wei L., Ma L., Wu Z., Gu C., Chen K. (2020). Comparative analyses of chloroplast genomes from 13 *Lagerstroemia* (Lythraceae) species: Identification of highly divergent regions and inference of phylogenetic relationships. Plant Mol. Biol..

[63] Hu Q. (1994). On the geographical distribution of the Primulaceae. J. Trop. Subtrop. Bot..

[64] Hao C.-Y., Tan L.-H., Fan R., Yu H., Yang J.-F., Wu H.-S. (2012). Floristic Geography of *Piper* (Piperaceae) in China. Plant Divers. Resour..

[65] Cui H.-X., Jiang G.-M., Zhang S.-Y. (2004). The distribution, origin and evolution of *Syringa*. Bull. Bot. Res..

[66] Yan H.F., Peng C.I., Hu C.M., Hao G. (2007). Phylogeographic structure of Primula obconica (Primulaceae) inferred from chloroplast microsatellites (cpSSRs) markers. Acta Phytotaxon. Sin..

[67] Yan H.F., Zhang C.Y., Wang F.Y., Hu C.M., Ge X.J., Hao G. (2012). Population Expanding with the Phalanx Model and Lineages Split by Environmental Heterogeneity: A Case Study of *Primula obconica* in Subtropical China. PLoS ONE.

[68] Zhang D.C., Boufford D.E., Ree R.H., Sun H. (2009). The 29° N latitudinal line: An important division in the Hengduan Mountains, a biodiversity hotspot in southwest China. Nord. J. Bot..

[69] Zhong L., Barrett S.C.H., Wang X.J., Wu Z.K., Sun H.Y., Li D.Z., Wang H., Zhou W. (2019). Phylogenomic analysis reveals multiple evolutionary origins of selfing from outcrossing in a lineage of heterostylous plants. New Phytol..

[70] Syring J., Farrell K., Businský R., Cronn R., Liston A. (2007). Widespread genealogical nonmonophyly in species of *Pinus* subgenus *Strobus*. Syst. Biol..

[71] McKay B.D., Zink R.M. (2010). The causes of mitochondrial DNA gene tree paraphyly in birds. Mol. Phylogenet. Evol..

[72] Schmidt-Lebuhn A.N., de Vos J.M., Keller B., Conti E. (2012). Phylogenetic analysis of *Primula* section *Primula* reveals rampant non-monophyly among morphologically distinct species. Mol. Phylogenet. Evol..

[73] Mast A.R., Feller D.M.S., Kelso S., Conti E. (2004). Buzz-pollinated Dodecatheon originated from within the heterostylous *Primula* subgenus *Auriculastrum* (Primulaceae): A seven-region cpDNA phylogeny and its implications for floral evolution. Am. J. Bot..

[74] Song L., Li Y., Zhang W., Shao J. (2017). Highly differentiated phylogeographic structure of *Primula ranunculoides*. Plant Sci. J..

[75] Wang F.Y., Ge X.J., Gong X., Hu C.M., Hao G. (2008). Strong genetic differentiation of *Primula sikkimensis* in the East Himalaya-Hengduan Mountains. Biochem. Genet..

[76] Tigano A., Friesen V.L. (2016). Genomics of local adaptation with gene flow. Mol. Ecol..

[77] Petit R.J., Excoffier L. (2009). Gene flow and species delimitation. Trends Ecol. Evol..

[78] Volis S., Ormanbekova D., Shulgina I. (2016). Fine-scale spatial genetic structure in predominantly selfing plants with limited seed dispersal: A rule or exception?. Plant Divers..

[79] Xue D., Ge X., Hao G., Zhang C. (2004). High genetic diversity in a rare, narrowly endemic primrose species: *Primula interjacens* by ISSR. Acta Bot. Sin..

[80] Van Geert A., Van Rossum F., Triest L. (2008). Genetic diversity in adult and seedling populations of *Primula vulgaris* in a fragmented agricultural landscape. Conserv. Genet..

[81] Kato J., Ohashi H., Ikeda M., Fujii N., Ishikawa R., Horaguchi H., Amano J., Hayashi M., Mii M. (2008). Unreduced gametes are the major causal factor for the production of polyploid interspecific hybrids in *Primula*. Plant Biotechnol..

[82] Ma Y.P., Tian X.L., Zhang J.L., Wu Z.K., Sun W.B. (2014). Evidence for natural hybridization between *Primula beesiana* and *P. bulleyana*, two heterostylous primroses in NW Yunnan, China. J. Syst. Evol..

[83] Qian C., Luo J., Mu J., Li C., Zhou Z., Ou M., Lan S. (2017). Studies on Pollination Insect Species and Their Visiting Behavior of *Primula lithophila* of Endemic Plants in Guizhou. Mol. Plant Breed..

[84] Abou-El-Enain M.M. (2006). Chromosomal variability in the genus *Primula* (Primulaceae). Bot. J. Linn. Soc..

[85] Kong H., Zhang Y., Hong Y., Barker M.S. (2017). Multilocus phylogenetic reconstruction informing polyploid relationships of *Aconitum* subgenus *Lycoctonum* (Ranunculaceae) in China. Plant Syst. Evol..

[86] Kim Y., Yi J.S., Min J., Xi H., Kim D.Y., Son J., Park J., Jeon J.I. (2019). The complete chloroplast genome of *Aconitum coreanum* (H. Lév.) Rapaics (Ranunculaceae). Mitochondrial DNA Part B Resour..

[87] Park I., Kim W.J., Yang S., Yeo S.M., Li H., Moon B.C. (2017). The complete chloroplast genome sequence of *Aconitum coreanum* and *Aconitum carmichaelii* and comparative analysis with other Aconitum species. PLoS ONE.

[88] Guggisberg A., Mansion G., Kelso S., Conti E. (2006). Evolution of biogeographic patterns, ploidy levels, and breeding systems in a diploid-polyploid species complex of *Primula*. New Phytol..

[89] Kong H.-Z., Liu J.-Q. (1999). Karyomorphology of the genus *Pomatosace* Maxin. (Primulaceae). Acta Phytotaxon. Sin..

[90] Zhu H.-F., Yang J.-B., Zhang C.-Q. (2002). Systematic position of *Primula secundiflora* (Primulaceae) inferred from nuclear ribosomal DNA ITS sequence data. Acta Phytotaxon. Sin..

[91] Xu Y., Choudhary R.K., Hao G., Hu C.M. (2020). *Primula subansirica* G.D. Pal is not a Primula (Primulaceae), but rather belongs to Gesneriaceae. Nord. J. Bot..

[92] Yi T.S., Jin G.H., Wen J. (2015). Chloroplast capture and intra- and inter-continental biogeographic diversification in the Asian—New World disjunct plant genus *Osmorhiza* (Apiaceae). Mol. Phylogenet. Evol..

[93] Casazza G., Granato L., Minuto L., Conti E. (2012). Polyploid evolution and Pleistocene glacial cycles: A case study from the alpine primrose *Primula marginata* (Primulaceae). BMC Evol. Biol..

[94] Cristina Acosta M., Premoli A.C. (2010). Evidence of chloroplast capture in South American Nothofagus (subgenus *Nothofagus*, Nothofagaceae). Mol. Phylogenet. Evol..

[95] Yan H.F., He C.H., Peng C.I., Hu C.M., Hao G. (2010). Circumscription of *Primula* subgenus *Auganthus* (Primulaceae) based on chloroplast DNA sequences. J. Syst. Evol..

[96] Liu Y.J., Liu J., Hu C.M., Hao G. (2015). Non-monophyly of *Primula* subgenera *Auganthus* and *Carolinella* (Primlaceae) as confirmed by the nuclear DNA sequence variation. Plant Syst. Evol..

[97] Ren T., Yang Y., Zhou T., Liu Z.L. (2018). Comparative plastid genomes of Primula species: Sequence divergence and phylogenetic relationships. Int. J. Mol. Sci..

[98] Trift I., Källersjö M., Anderberg A.A. (2002). The monophyly of *Primula* (Primulaceae) evaluated by analysis of sequences from the chloroplast gene rbcL. Syst. Bot..

[99] Mast A.R., Kelso S., Conti E. (2006). Are any primroses (*Primula*) primitively monomorphic?. New Phytol..

[100] Ren G., Conti E., Salamin N. (2015). Phylogeny and biogeography of *Primula* sect. *Armerina*: Implications for plant evolution under climate change and the uplift of the Qinghai-Tibet Plateau. BMC Evol. Biol..

[101] Conti E., Suring E., Boyd D., Jorgensen J., Grant J., Kelso S. (2000). Phylogenetic relationships and character evolution in *primula* L.: The usefulness of ITS sequence data. Plant Biosyst..

[102] Hao G., Hu C.M., Lee N.S. (2002). Circumscriptions and phylogenetic relationships of *Primula* sects. *Auganthus* and *Ranunculoides*: Evidence from nrDNA ITS sequences. Acta Bot. Sin..

