# Supplementary material for: The Complete Chloroplast Genomes of Primula obconica Provide Insight That Neither Species nor Natural Section Represent Monophyletic Taxa in Primula (Primulaceae)
Source: Genes (Basel). 2022 Mar 23;13(4):567. doi: 10.3390/genes13040567 (PMC9030805; doi:10.3390/genes13040567)
Supplement: Supplementary file 1 [file genes-13-00567-s001.zip › Supplemental Table S1.pdf]

Table S1. Details of accession used in the study.

Genbank accession numbers	Accession Id	Origin	Coordinates
OK040681	HBD1	Hubei, China	30.9672N 110.6137E
OK040682	HBD2	Hubei, China	30.9672N 110.6137E
OK040683	HBE1	Hubei, China	29.829N 109.5805E
OK040684	HBE2	Hubei, China	29.829N 109.5805E
OK040687	HNL1	Hunan, China	28.3704N 109.2923E
OK040688	HNL2	Hunan, China	28.3704N 109.2923E
OK040685	HND1	Hunan, China	28.3425N 109.2803E
OK040686	HND2	Hunan, China	28.3425N 109.2803E
OK040690	ZP1	Anhui, China,	cultivated
OK040689	ZP2	Anhui, China,	cultivated
OK040691	SCB1	Sichuan, China	30.4205N 102.6529E
OK040692	SCB2	Sichuan, China	30.4205N 102.6529E
OK040693	SCD1	Sichuan, China	31.0819N 103.5757E
OK040694	SCD2	Sichuan, China	31.0819N 103.5757E
OK040695	YN1	Yunnan, China	27.3893N 104.8214E
OK040696	YN2	Yunnan, China	27.3893N 104.8214E

IBSC, South China Botanical Garden
