# Supplementary material for: The Complete Chloroplast Genomes of Primula obconica Provide Insight That Neither Species nor Natural Section Represent Monophyletic Taxa in Primula (Primulaceae)
Source: Genes (Basel). 2022 Mar 23;13(4):567. doi: 10.3390/genes13040567 (PMC9030805; doi:10.3390/genes13040567)
Supplement: Supplementary file 1 [file genes-13-00567-s001.zip › Supplemental Table S2.pdf]

Table S2. Comparison of general characteristics of the cp genomes of *P. obconica* subsp. *obconica*.

Species	Total size(bp)	GC content(%)	LSC size(bp)	SSC size(bp)	IR size(bp)	number of genes	rRNA gene number	tRNA gene number
HBD1	153861	37.2	84599	17676	25793	128	37	8
HBD2	153861	37.2	84599	17676	25793	128	37	8
HNL2	154028	37.1	84701	17743	25792	128	37	8
HNL2	154028	37.1	84701	17743	25792	128	37	8
HND1	154028	37.1	84701	17743	25792	128	37	8
HND2	154028	37.1	84701	17743	25792	128	37	8
YN1	153645	37.3	84403	17690	25776	128	37	8
YN2	153604	37.3	84404	17690	25755	128	37	8
HBE1	153883	37.2	84537	17718	25814	128	37	8
HBE2	153883	37.2	84537	17718	25814	128	37	8
ZP2	153954	37.2	84628	17740	25793	128	37	8
ZP1	153837	37.2	84587	17658	25796	128	37	8
SCD1	153591	37.3	84712	17347	25766	128	37	8
SCD2	153591	37.3	84712	17347	25766	128	37	8
SCB1	153584	37.3	84692	17360	25766	128	37	8
SCB2	153584	37.3	84692	17360	25766	128	37	8
