# Supplementary material for: The Complete Chloroplast Genomes of Primula obconica Provide Insight That Neither Species nor Natural Section Represent Monophyletic Taxa in Primula (Primulaceae)
Source: Genes (Basel). 2022 Mar 23;13(4):567. doi: 10.3390/genes13040567 (PMC9030805; doi:10.3390/genes13040567)
Supplement: Supplementary file 1 [file genes-13-00567-s001.zip › Supplemental Table S3.pdf]

Table S3. List of genes in the chloroplast genomes of *P. obconica* subsp. *obconica*

Category	Group of gene	Gene name
Genes for photosynthesis	Subunits of ATP synthase	*atpA, atpB, atpE, atpF, atpH, atpI*
	Subunits of photosystem II	*psbA, psbB, psbC, psbD, psbE, psbF, psbI, psbJ, psbK, psbL, psbM, psbN, psbT, psbZ, ycf3*
	Subunits of NADH-dehydrogenase	*ndhA, ndhB, ndhB, ndhC, ndhD, ndhE, ndhF, ndhG, ndhH, ndhI, ndhJ, ndhK*
	Subunits of cytochrome b/f complex	*petA, petB, petD, petG, petL, petN*
	Subunits of photosystem I	*psaA, psaB, psaC, psaI, psaJ*
	Subunit of rubisco	*rbcL*
Self replication	Large subunit of ribosome	*rpl14, rpl16, rpl2, rpl2, rpl20, rpl22, rpl23, rpl23, rpl32, rpl33, rpl36*
	DNA dependent RNA polymerase	*rpoA, rpoB, rpoC1, rpoC2*
	Small subunit of ribosome	*rps11, rps12, rps12, rps14, rps15, rps16, rps18, rps19, rps2, rps3, rps4, rps7, rps7, rps8*
Other genes	c-type cytochrom synthesis gene	*ccsA*
	Envelop membrane protein	*cemA*
	Protease	*clpP*
	Maturase	*matK*
Unkown	Conserved open reading frames	*ycf1, ycf2, ycf2, ycf4*
