# Supplementary material for: The Complete Chloroplast Genomes of Primula obconica Provide Insight That Neither Species nor Natural Section Represent Monophyletic Taxa in Primula (Primulaceae)
Source: Genes (Basel). 2022 Mar 23;13(4):567. doi: 10.3390/genes13040567 (PMC9030805; doi:10.3390/genes13040567)
Supplement: Supplementary file 1 [file genes-13-00567-s001.zip › Supplemental Table S4.pdf]

Table S4. The lengths of introns and exons for the splitting genes in *P. obconica* subsp. *obconica*

Species	Gene	Start	End	Exon I (bp)	Intron I (bp)	Exon II (bp)	Intron II (bp)	Exon III (bp)
HBD	trnK-UUU	1766	4323	37	2486	35		
	rps16	5000	6112	40	840	233		
	trnG-UCC	8990	9749	23	689	48		
	atpF	11788	13064	145	722	410		
	rpoC1	20547	23354	432	759	1617		
	ycf3	42142	44077	126	715	228	714	153
	trnL-UAA	47078	47677	35	515	50		
	trnV-UAC	51593	52269	39	603	35		
	clpP	70300	72379	71	849	294	640	226
	petB	75326	76740	6	767	642		
	petD	76934	78155	8	739	475		
	rpl16	81616	83023	9	1000	399		
	rpl2	84661	86149	394	661	434		
	ndhB	94960	97171	775	679	758		
	trnI-GAU	102755	103585	37	759	35		
	trnA-UGC	103649	104531	38	810	35		
	ndhF	109608	112577	1781	1188	1		
	ndhA	119424	121530	469	1099	539		
	trnA-UGC	133930	134812	38	810	35		
	trnI-GAU	134876	135706	37	759	35		
	ndhB	141290	143501	775	679	758		
	rpl2	152312	153800	394	661	434		
HBE	trnK-UUU	1766	4324	37	2487	35		
	rps16	5001	6100	40	827	233		
	trnG-UCC	8978	9737	23	689	48		
	atpF	11777	13064	145	733	410		
	rpoC1	20547	23352	432	757	1617		
	ycf3	42149	44088	126	713	228	720	153
	trnL-UAA	47076	47674	35	514	50		
	trnV-	51602	52278	39	603	35		

	UAC						
	clpP	70220	72300	71	850	294	640	226
	petB	75247	76662	6	768	642		
	petD	76856	78095	8	757	475		
	rpl16	81558	82961	9	996	399		
	rpl2	84599	86087	394	661	434		
	ndhB	94919	97130	775	679	758		
	trnI-GAU	102714	103544	37	759	35		
	trnA-UGC	103608	104490	38	810	35		
	ndhF	109567	112536	1781	1188	1		
	ndhA	119464	121530	469	1059	539		
	trnA-UGC	133931	134813	38	810	35		
	trnI-GAU	134877	135707	37	759	35		
	ndhB	141291	143502	775	679	758		
	rpl2	152334	153822	394	661	434		
HND	trnK-UUU	1766	4325	37	2488	35		
	rps16	5002	6111	40	837	233		
	trnG-UCC	8990	9749	23	689	48		
	atpF	11787	13074	145	733	410		
	rpoC1	20560	23376	432	768	1617		
	ycf3	42195	44135	126	714	228	720	153
	trnL-UAA	47166	47764	35	514	50		
	trnV-UAC	51679	52355	39	603	35		
	clpP	70358	72436	71	848	294	640	226
	petB	75383	76798	6	768	642		
	petD	76992	78268	8	794	475		
	rpl16	81729	83137	9	1001	399		
	rpl2	84763	86251	394	661	434		
	ndhB	95062	97273	775	679	758		
	trnI-GAU	102857	103687	37	759	35		
	trnA-UGC	103751	104632	38	809	35		
	ndhF	109709	112678	1781	1188	1		
	ndhA	119591	121697	469	1099	539		
	trnA-	134098	134979	38	809	35		

	UGC							
	trnI-GAU	135043	135873	37	759	35		
	ndhB	141457	143668	775	679	758		
	rpl2	152479	153967	394	661	434		
HNL	trnK-UUU	1766	4325	37	2488	35		
	rps16	5002	6111	40	837	233		
	trnG-UCC	8990	9749	23	689	48		
	atpF	11787	13074	145	733	410		
	rpoC1	20560	23376	432	768	1617		
	ycf3	42195	44135	126	714	228	720	153
	trnL-UAA	47166	47764	35	514	50		
	trnV-UAC	51679	52355	39	603	35		
	clpP	70358	72436	71	848	294	640	226
	petB	75383	76798	6	768	642		
	petD	76992	78268	8	794	475		
	rpl16	81729	83137	9	1001	399		
	rpl2	84763	86251	394	661	434		
	ndhB	95062	97273	775	679	758		
	trnI-GAU	102857	103687	37	759	35		
	trnA-UGC	103751	104632	38	809	35		
	ndhF	109709	112678	1781	1188	1		
	ndhA	119591	121697	469	1099	539		
	trnA-UGC	134098	134979	38	809	35		
	trnI-GAU	135043	135873	37	759	35		
	ndhB	141457	143668	775	679	758		
	rpl2	152479	153967	394	661	434		
ZP1	trnK-UUU	1766	4326	37	2489	35		
	rps16	5003	6112	40	837	233		
	trnG-UCC	8992	9751	23	689	48		
	atpF	11790	13077	145	733	410		
	rpoC1	20560	23366	432	758	1617		
	ycf3	42183	44117	126	714	228	714	153
	trnL-	47147	47747	35	516	50		

	UAA							
	trnV-UAC	51599	52275	39	603	35		
	clpP	70288	72367	71	850	294	639	226
	petB	75314	76728	6	767	642		
	petD	76922	78143	8	739	475		
	rpl16	81605	83011	9	999	399		
	rpl2	84649	86137	394	661	434		
	ndhB	94948	97159	775	679	758		
	trnI-GAU	102744	103574	37	759	35		
	trnA-UGC	103638	104522	38	812	35		
	ndhF	109599	112568	1781	1188	1		
	ndhA	119396	121502	469	1099	539		
	trnA-UGC	133903	134787	38	812	35		
	trnI-GAU	134851	135681	37	759	35		
	ndhB	141266	143477	775	679	758		
	rpl2	152288	153776	394	661	434		
ZP2	trnK-UUU	1766	4324	37	2487	35		
	rps16	5001	6113	40	840	233		
	trnG-UCC	8993	9752	23	689	48		
	atpF	11792	13068	145	722	410		
	rpoC1	20563	23369	432	758	1617		
	ycf3	42150	44084	126	714	228	714	153
	trnL-UAA	47116	47714	35	514	50		
	trnV-UAC	51628	52304	39	603	35		
	clpP	70328	72407	71	850	294	639	226
	petB	75354	76769	6	768	642		
	petD	76963	78184	8	739	475		
	rpl16	81645	83052	9	1000	399		
	rpl2	84690	86178	394	661	434		
	ndhB	94989	97200	775	679	758		
	trnI-GAU	102784	103615	37	760	35		
	trnA-UGC	103679	104560	38	809	35		
	ndhF	109637	112606	1781	1188	1		

	ndhA	119516	121622	469	1099	539		
	trnA-UGC	134023	134904	38	809	35		
	trnI-GAU	134968	135799	37	760	35		
	ndhB	141383	143594	775	679	758		
	rpl2	152405	153893	394	661	434		
SCB	trnK-UUU	1782	4342	37	2489	35		
	rps16	4997	6085	40	816	233		
	trnG-UCC	8955	9714	23	689	48		
	atpF	11753	13034	145	727	410		
	rpoC1	20642	23435	432	745	1617		
	ycf3	42435	44370	124	713	230	716	153
	trnL-UAA	47416	48013	35	513	50		
	trnV-UAC	51924	52593	39	596	35		
	clpP	70486	72576	71	854	294	646	226
	petB	75523	76937	6	767	642		
	petD	77131	78399	8	786	475		
	rpl16	81868	83116	9	841	399		
	rpl2	84754	86242	394	661	434		
	ndhB	95053	97264	775	679	758		
	trnI-GAU	102854	103684	37	759	35		
	trnA-UGC	103748	104629	38	809	35		
	ndhF	109661	112635	1781	1193	1		
	ndhA	119212	121313	469	1094	539		
	trnA-UGC	133648	134529	38	809	35		
	trnI-GAU	134593	135423	37	759	35		
	ndhB	141013	143224	775	679	758		
	rpl2	152035	153523	394	661	434		
SCD	trnK-UUU	1766	4326	37	2489	35		
	rps16	4888	5998	40	838	233		
	trnG-UCC	8861	9620	23	689	48		
	atpF	11661	12934	145	719	410		
	rpoC1	20543	23336	432	745	1617		

	ycf3	42331	44272	124	715	230	720	153
	trnL-UAA	47305	47901	35	512	50		
	trnV-UAC	51806	52475	39	596	35		
	clpP	70329	72419	71	854	294	646	226
	petB	75366	76780	6	767	642		
	petD	76974	78254	8	798	475		
	rpl16	81724	83136	9	1005	399		
	rpl2	84774	86262	394	661	434		
	ndhB	95073	97284	775	679	758		
	trnI-GAU	102874	103704	37	759	35		
	trnA-UGC	103768	104649	38	809	35		
	ndhF	109681	112655	1781	1193	1		
	ndhA	119225	121326	469	1094	539		
	trnA-UGC	133655	134536	38	809	35		
	trnI-GAU	134600	135430	37	759	35		
	ndhB	141020	143231	775	679	758		
	rpl2	152042	153530	394	661	434		
YN	trnK-UUU	1767	4325	37	2487	35		
	rps16	4996	6105	40	837	233		
	trnG-UCC	8978	9737	23	689	48		
	atpF	11789	13051	145	720	398		
	rpoC1	20646	23439	432	745	1617		
	ycf3	42085	44026	124	715	230	720	153
	trnL-UAA	47056	47653	35	513	50		
	trnV-UAC	51563	52232	39	596	35		
	clpP	70056	72139	71	847	294	646	226
	petB	75079	76493	6	767	642		
	petD	76687	77949	8	780	475		
	rpl16	81416	82827	9	1004	399		
	rpl2	84465	85953	394	661	434		
	ndhB	94785	96996	775	679	758		
	trnI-GAU	102586	103416	37	759	35		
	trnA-	103480	104361	38	809	35		

UGC					
ndhF	109388	112362	1781	1193	1
ndhA	119251	121352	469	1094	539
trnA-UGC	133688	134569	38	809	35
trnI-GAU	134633	135463	37	759	35
ndhB	141053	143264	775	679	758
rpl2	152096	153584	394	661	434
