# Supplementary material for: The Complete Chloroplast Genomes of Primula obconica Provide Insight That Neither Species nor Natural Section Represent Monophyletic Taxa in Primula (Primulaceae)
Source: Genes (Basel). 2022 Mar 23;13(4):567. doi: 10.3390/genes13040567 (PMC9030805; doi:10.3390/genes13040567)
Supplement: Supplementary file 1 [file genes-13-00567-s001.zip › Supplemental Table S5.pdf]

Table S5: SNPs identified in *P. obconica* subsp. *obconica* chloroplast genomes

SNP position	Ref	Alt	Location
135	A	C	psbA
200	T	G	psbA
225	G	A	psbA
311	G	A	psbA
342	C	T	psbA
372	A	C	psbA
375	C	A	psbA
409	T	G	psbA
426	T	C	psbA
865	A	G	CDS
889	G	A	CDS
1021	G	A	psbA
1153	A	G	psaA
1504	A	G	psaA
1537	A	G	psbA
1636	C	G	psbA-matK
1666	T	G	psbA-matK
1708	A	T	psbA-matK
1714	A	C	psbA-matK
1748	C	A	psbA-matK
1762	T	G	psbA-matK
1865	A	G	psbA-matK
1888	T	G	psbA-matK
1907	C	T	psbA-matK
1933	G	T	psbA-matK
1934	A	C	psbA-matK
1940	T	G	psbA-matK
1945	T	G	psbA-matK
1946	C	A	psbA-matK
1966	A	C	psbA-matK
1980	G	A	psbA-matK
1982	A	C	psbA-matK
2040	A	G	psbA-matK
2161	C	A	matK
2199	A	G	matK
2203	A	T	matK
2226	T	C	matK
2269	C	T	matK
2310	A	G	matK
2368	T	C	matK
2382	C	A	matK

2394	T	C	matK
2440	A	G	matK
2445	T	C	matK
2466	T	C	matK
2536	C	A	matK
2791	G	A	matK
2832	T	G	matK
3033	T	G	matK
3045	C	T	matK
3056	C	A	matK
3073	C	T	matK
3143	G	A	matK
3217	T	C	matK
3222	T	G	matK
3284	T	G	matK
3297	C	T	matK
3366	T	C	matK
3460	C	T	matK
3462	C	G	matK
3515	G	T	matK
3519	C	T	matK
3546	G	T	matK
3586	T	C	matK
3601	T	G	matK-rps16
3633	G	A	matK-rps16
3661	C	A	matK-rps16
3702	G	A	matK-rps16
3748	T	A	matK-rps16
3749	A	C	matK-rps16
3793	C	A	matK-rps16
3821	G	A	matK-rps16
3864	T	C	matK-rps16
3917	C	A	matK-rps16
3958	T	C	matK-rps16
4021	C	T	matK-rps16
4040	C	T	matK-rps16
4108	G	A	matK-rps16
4111	C	T	matK-rps16
4127	A	C	matK-rps16
4289	G	A	matK-rps16
4290	C	T	matK-rps16
4408	G	A	matK-rps16
4455	A	C	matK-rps16
4460	A	C	matK-rps16

4462	T	C	matK-rps16
4483	T	C	matK-rps16
4510	A	C	matK-rps16
4586	A	T	matK-rps16
4597	G	A	matK-rps16
4614	A	G	matK-rps16
4614	A	T	matK-rps16
4620	A	C	matK-rps16
4699	T	G	matK-rps16
4703	T	G	matK-rps16
4706	A	G	matK-rps16
4708	C	A	matK-rps16
4749	G	A	matK-rps16
4788	G	T	matK-rps16
4843	C	T	matK-rps16
4872	T	G	matK-rps16
4881	G	A	matK-rps16
4893	G	A	matK-rps16
4906	T	G	matK-rps16
4913	T	C	matK-rps16
4946	C	A	matK-rps16
4956	T	C	matK-rps16
4957	T	G	matK-rps16
4978	T	G	matK-rps16
4992	G	T	rps16
5060	C	G	rps16
5127	A	C	rps16
5240	G	T	rps16
5311	G	T	rps16
5316	G	T	rps16
5366	G	T	rps16
5389	T	C	rps16
5398	A	T	rps16
5491	G	T	rps16
5495	C	T	rps16
5523	T	C	rps16
5650	C	G	rps16
5748	G	T	rps16
5763	C	T	rps16
5869	C	T	rps16
5989	C	T	rps16
6180	T	G	rps16-psbK
6184	C	G	rps16-psbK
6208	A	C	rps16-psbK

6244	T	C	rps16-psbK
6278	G	A	rps16-psbK
6288	A	C	rps16-psbK
6330	A	G	rps16-psbK
6345	T	G	rps16-psbK
6442	A	G	rps16-psbK
6479	T	G	rps16-psbK
6527	C	T	rps16-psbK
6554	G	A	rps16-psbK
6611	A	G	rps16-psbK
6629	G	A	rps16-psbK
6650	G	A	rps16-psbK
6731	C	T	rps16-psbK
6740	C	T	rps16-psbK
6780	T	G	rps16-psbK
6790	T	C	rps16-psbK
6800	A	C	rps16-psbK
6806	A	C	rps16-psbK
6813	A	C	rps16-psbK
6825	C	A	rps16-psbK
6826	G	A	rps16-psbK
6844	A	C	rps16-psbK
6925	T	A	rps16-psbK
6967	T	C	rps16-psbK
6972	G	A	rps16-psbK
6983	T	G	rps16-psbK
6998	T	G	rps16-psbK
7093	C	T	rps16-psbK
7117	A	G	rps16-psbK
7161	T	G	rps16-psbK
7172	T	G	rps16-psbK
7206	C	A	rps16-psbK
7226	A	C	rps16-psbK
7233	A	C	rps16-psbK
7281	T	C	rps16-psbK
7347	C	A	rps16-psbK
7348	C	A	rps16-psbK
7353	T	G	rps16-psbK
7445	T	C	psbK
7485	A	C	psbK
7496	C	T	psbK
7511	G	A	psbK
7732	A	C	psbK-psbI
7741	A	C	psbK-psbI

7748	G	T	psbK-psbI
7817	A	C	psbK-psbI
7835	C	A	psbK-psbI
7843	G	A	psbK-psbI
7883	C	T	psbK-psbI
7900	C	T	psbK-psbI
7937	G	A	psbK-psbI
7952	G	A	psbK-psbI
7966	G	A	psbK-psbI
7994	G	T	psbK-psbI
7995	G	T	psbK-psbI
8165	A	C	psbI-atpA
8180	T	C	psbI-atpA
8355	T	G	psbI-atpA
8391	A	C	psbI-atpA
8469	G	C	psbI-atpA
8523	G	A	psbI-atpA
8542	A	C	psbI-atpA
8572	G	T	psbI-atpA
8622	A	C	psbI-atpA
8622	A	T	psbI-atpA
8649	G	T	psbI-atpA
8656	A	C	psbI-atpA
8669	A	C	psbI-atpA
8670	T	G	psbI-atpA
8697	T	G	psbI-atpA
8771	T	C	psbI-atpA
8772	A	G	psbI-atpA
8772	A	C	psbI-atpA
8773	A	G	psbI-atpA
8808	A	C	psbI-atpA
8851	G	T	psbI-atpA
8862	C	T	psbI-atpA
8879	C	T	psbI-atpA
8885	G	A	psbI-atpA
8886	G	T	psbI-atpA
9022	T	C	psbI-atpA
9070	T	A	psbI-atpA
9126	T	G	psbI-atpA
9134	G	A	psbI-atpA
9192	A	C	psbI-atpA
9275	A	G	psbI-atpA
9275	A	T	psbI-atpA
9289	G	A	psbI-atpA

9326	A	C	psbI-atpA
9342	G	A	psbI-atpA
9347	A	G	psbI-atpA
9366	A	G	psbI-atpA
9405	A	T	psbI-atpA
9503	A	C	psbI-atpA
9532	G	T	psbI-atpA
9569	C	A	psbI-atpA
9628	A	C	psbI-atpA
9640	T	C	psbI-atpA
9648	G	T	psbI-atpA
9726	T	C	psbI-atpA
9776	C	T	psbI-atpA
9801	A	G	psbI-atpA
9857	T	C	psbI-atpA
9894	G	T	psbI-atpA
9899	A	C	psbI-atpA
9971	T	C	psbI-atpA
9985	C	A	psbI-atpA
10066	G	A	psbI-atpA
10143	T	C	psbI-atpA
10286	G	A	atpA
10292	A	C	atpA
10298	A	C	atpA
10478	C	T	atpA
10511	T	G	atpA
10538	C	T	atpA
10657	G	A	atpA
11282	T	C	atpA
11334	G	A	atpA
11341	T	G	atpA
11423	G	A	atpA
11615	T	A	atpA
11648	C	T	atpA
11718	G	C	atpA-atpF
11769	C	A	atpF
11840	T	G	atpF
11888	C	T	atpF
11892	C	T	atpF
11987	A	C	atpF
11996	C	T	atpF
12043	G	T	atpF
12089	G	A	atpF
12097	G	A	atpF

12194	C	T	atpF
12475	C	A	atpF
12526	T	C	atpF
12587	A	C	atpF
12588	G	A	atpF
12613	G	A	atpF
12726	G	A	atpF
12753	A	G	atpF
12767	T	G	atpF
12773	A	C	atpF
12840	T	C	atpF
12844	A	G	atpF
12907	T	G	atpF
12939	G	A	atpF
12980	A	G	atpF
13092	A	C	atpF-atpH
13145	G	T	atpF-atpH
13149	T	A	atpF-atpH
13211	A	G	atpF-atpH
13330	C	T	atpF-atpH
13388	A	G	atpF-atpH
13401	A	C	atpF-atpH
13408	A	C	atpF-atpH
13567	A	G	atpH
13591	T	C	atpH
13597	T	C	atpH
13751	T	C	atpH-atpI
13809	T	C	atpH-atpI
13849	A	C	atpH-atpI
13930	A	C	atpH-atpI
13948	T	C	atpH-atpI
13995	C	T	atpH-atpI
14044	T	C	atpH-atpI
14137	C	T	atpH-atpI
14204	C	A	atpH-atpI
14207	T	C	atpH-atpI
14547	A	G	atpI
14775	G	A	atpI
14892	A	G	atpI
14940	C	T	atpI
14958	G	T	atpI
14985	C	G	atpI
15209	G	T	atpI-rps2
15214	A	C	atpI-rps2

15222	T	G	atpI-rps2
15287	C	A	atpI-rps2
15312	C	T	atpI-rps2
15330	C	A	atpI-rps2
15396	A	C	rps2
15588	T	C	rps2
15661	T	C	rps2
15672	T	G	rps2
15703	A	G	rps2
15794	T	C	rps2
15797	G	A	rps2
15967	C	T	rps2
16038	G	T	rps2-rpoC2
16055	C	T	rps2-rpoC2
16103	A	G	rps2-rpoC2
16160	T	G	rps2-rpoC2
16178	A	G	rps2-rpoC2
16209	G	T	rps2-rpoC2
16284	G	T	rps2-rpoC2
16422	G	T	rpoC2
16618	G	A	rpoC2
16696	T	C	rpoC2
16744	A	G	rpoC2
16822	G	A	rpoC2
16828	C	T	rpoC2
16896	G	A	rpoC2
17020	T	C	rpoC2
17137	T	C	rpoC2
17441	G	T	rpoC2
17453	G	A	rpoC2
17459	G	T	rpoC2
17480	G	A	rpoC2
17489	T	A	rpoC2
17496	T	G	rpoC2
17530	C	T	rpoC2
17561	G	T	rpoC2
17729	A	G	rpoC2
17743	G	A	rpoC2
18034	C	T	rpoC2
18040	A	C	rpoC2
18148	C	T	rpoC2
18182	C	A	rpoC2
18202	C	T	rpoC2
18341	C	T	rpoC2

18380	C	A	rpoC2
18432	G	A	rpoC2
18479	G	A	rpoC2
18544	C	T	rpoC2
18748	C	T	rpoC2
18763	C	G	rpoC2
18843	T	C	rpoC2
18911	T	G	rpoC2
18956	T	G	rpoC2
19016	A	G	rpoC2
19168	C	A	rpoC2
19361	A	C	rpoC2
19405	C	A	rpoC2
19735	G	T	rpoC2
19743	A	T	rpoC2
19903	A	G	rpoC2
20191	C	T	rpoC2
20284	T	G	rpoC2
20486	T	C	rpoC2-rpoC1
20587	A	C	rpoC2-rpoC1
20618	T	C	rpoC2-rpoC1
21029	C	T	rpoC1
21123	A	C	rpoC1
21237	A	G	rpoC1
21462	T	C	rpoC1
21471	T	C	rpoC1
21510	C	A	rpoC1
21693	C	T	rpoC1
21746	A	C	rpoC1
21753	G	A	rpoC1
21864	T	G	rpoC1
21912	A	C	rpoC1
21951	C	T	rpoC1
22034	A	T	rpoC1
22037	C	T	rpoC1
22038	A	C	rpoC1
22043	T	G	rpoC1
22131	G	T	rpoC1
22149	G	A	rpoC1
22342	A	G	rpoC1
22483	C	T	rpoC1
22498	G	T	rpoC1
22717	G	T	rpoC1
22767	A	G	rpoC1

22790	A	C	rpoC1
22829	C	T	rpoC1
22898	T	G	rpoC1
22918	A	G	rpoC1
22973	C	T	rpoC1
23045	T	G	rpoC1
23262	C	T	rpoC1
23667	G	A	rpoB
23678	C	G	rpoB
23826	C	T	rpoB
23896	T	G	rpoB
24027	T	C	rpoB
24189	C	T	rpoB
24216	G	T	rpoB
24221	C	T	rpoB
24375	A	G	rpoB
24579	A	C	rpoB
24627	C	T	rpoB
24738	A	G	rpoB
25083	C	T	rpoB
25095	A	G	rpoB
25142	C	A	rpoB
25168	C	A	rpoB
25200	C	T	rpoB
25207	A	G	rpoB
25212	T	C	rpoB
25228	T	G	rpoB
25281	C	T	rpoB
25470	C	T	rpoB
25488	G	A	rpoB
25632	T	C	rpoB
25761	C	T	rpoB
25769	T	G	rpoB
25841	C	T	rpoB
25855	C	T	rpoB
25904	G	A	rpoB
25949	G	A	rpoB
26011	A	T	rpoB
26223	A	G	rpoB
26400	T	G	rpoB
26565	T	C	rpoB
26572	T	G	rpoB
26613	A	C	rpoB
26652	G	T	rpoB

26755	G	A	rpoB-petN
26771	T	G	rpoB-petN
26798	T	G	rpoB-petN
26839	A	C	rpoB-petN
26881	T	G	rpoB-petN
26888	G	T	rpoB-petN
26965	G	A	rpoB-petN
27027	T	C	rpoB-petN
27045	G	T	rpoB-petN
27050	C	A	rpoB-petN
27057	T	G	rpoB-petN
27205	G	A	rpoB-petN
27308	T	C	rpoB-petN
27324	A	C	rpoB-petN
27324	A	G	rpoB-petN
27359	G	A	rpoB-petN
27459	G	A	rpoB-petN
27529	A	C	rpoB-petN
27538	G	T	rpoB-petN
27543	C	A	rpoB-petN
27597	T	G	rpoB-petN
27605	A	G	rpoB-petN
27609	C	T	rpoB-petN
27693	T	G	rpoB-petN
27758	T	G	rpoB-petN
27762	C	T	rpoB-petN
27879	A	C	rpoB-petN
27902	A	G	rpoB-petN
27903	G	A	rpoB-petN
27991	A	G	rpoB-petN
28009	A	G	rpoB-petN
28014	G	T	rpoB-petN
28028	T	C	rpoB-petN
28091	G	A	rpoB-petN
28139	T	G	rpoB-petN
28244	C	T	rpoB-petN
28354	G	T	petN-psbM
28370	A	G	petN-psbM
28414	C	T	petN-psbM
28425	A	C	petN-psbM
28492	G	A	petN-psbM
28509	A	C	petN-psbM
28520	T	G	petN-psbM
28557	C	T	petN-psbM

28563	C	G	petN-psbM
28739	C	T	petN-psbM
28786	A	C	petN-psbM
28827	T	G	petN-psbM
28862	G	A	petN-psbM
28877	A	C	petN-psbM
28924	G	A	petN-psbM
28926	A	C	petN-psbM
28927	C	A	petN-psbM
29007	C	T	petN-psbM
29007	C	A	petN-psbM
29023	A	G	petN-psbM
29040	C	T	petN-psbM
29071	T	C	petN-psbM
29091	G	A	petN-psbM
29175	T	C	petN-psbM
29177	C	T	petN-psbM
29189	G	A	petN-psbM
29195	G	A	petN-psbM
29223	G	A	petN-psbM
29350	C	T	psbM
29375	T	A	psbM-psbD
29375	T	C	psbM-psbD
29379	T	A	psbM-psbD
29637	C	T	psbM-psbD
29653	G	A	psbM-psbD
29707	C	T	psbM-psbD
29749	G	A	psbM-psbD
29767	A	G	psbM-psbD
29767	A	C	psbM-psbD
29839	T	G	psbM-psbD
29846	T	C	psbM-psbD
29875	T	G	psbM-psbD
29881	T	C	psbM-psbD
29942	A	G	psbM-psbD
29973	A	G	psbM-psbD
30025	A	C	psbM-psbD
30038	G	T	psbM-psbD
30048	G	A	psbM-psbD
30088	G	A	psbM-psbD
30142	T	G	psbM-psbD
30149	G	A	psbM-psbD
30162	T	C	psbM-psbD
30181	T	G	psbM-psbD

30203	G	T	psbM-psbD
30211	T	G	psbM-psbD
30245	A	G	psbM-psbD
30267	C	T	psbM-psbD
30323	C	A	psbM-psbD
30350	T	G	psbM-psbD
30369	G	A	psbM-psbD
30453	A	G	psbM-psbD
30585	A	G	psbM-psbD
30593	T	G	psbM-psbD
30598	A	G	psbM-psbD
30654	C	G	psbM-psbD
30668	G	T	psbM-psbD
30909	T	C	psbM-psbD
30982	A	C	psbM-psbD
31019	G	C	psbM-psbD
31021	T	A	psbM-psbD
31030	C	G	psbM-psbD
31041	C	T	psbM-psbD
31044	T	C	psbM-psbD
31051	G	T	psbM-psbD
31062	G	T	psbM-psbD
31068	G	A	psbM-psbD
31089	T	C	psbM-psbD
31093	A	T	psbM-psbD
31093	A	G	psbM-psbD
31096	A	C	psbM-psbD
31108	A	G	psbM-psbD
31111	C	A	psbM-psbD
31116	C	A	psbM-psbD
31130	T	A	psbM-psbD
31141	A	C	psbM-psbD
31149	T	G	psbM-psbD
31170	C	T	psbM-psbD
31198	C	T	psbM-psbD
31208	G	A	psbM-psbD
31216	A	T	psbM-psbD
31217	T	C	psbM-psbD
31220	C	A	psbM-psbD
31228	A	T	psbM-psbD
31238	T	C	psbM-psbD
31251	G	A	psbM-psbD
31286	G	T	psbM-psbD
31289	A	C	psbM-psbD

31291	A	C	psbM-psbD
31303	T	C	psbM-psbD
31306	G	A	psbM-psbD
31336	T	A	psbM-psbD
31401	T	G	psbM-psbD
31500	A	G	psbM-psbD
31523	C	T	psbM-psbD
31562	A	C	psbM-psbD
31699	A	C	psbM-psbD
31723	C	A	psbM-psbD
31729	A	G	psbM-psbD
31738	T	C	psbM-psbD
31779	A	G	psbM-psbD
31825	G	T	psbM-psbD
31844	C	T	psbM-psbD
31887	C	T	psbM-psbD
31902	T	G	psbM-psbD
31925	A	G	psbM-psbD
31926	T	G	psbM-psbD
31982	C	T	psbM-psbD
32007	A	G	psbM-psbD
32045	T	G	psbM-psbD
32163	A	C	psbM-psbD
32203	C	T	psbM-psbD
32223	A	C	psbM-psbD
32247	A	C	psbM-psbD
32298	C	A	psbM-psbD
32308	A	C	psbM-psbD
32373	G	T	psbM-psbD
32374	A	C	psbM-psbD
32377	C	T	psbM-psbD
32430	G	A	psbM-psbD
32460	G	A	psbM-psbD
32570	G	A	psbM-psbD
32591	G	T	psbM-psbD
32750	G	A	psbM-psbD
33068	T	C	psbD
33104	C	T	psbD
33218	T	C	psbD
33219	C	T	psbD
33917	T	C	psbD-psbC
33990	T	C	psbC
34047	C	A	psbC
34137	T	G	psbC

34167	C	T	psbC
34239	T	C	psbC
34308	T	C	psbC
34494	T	G	psbC
34567	A	G	psbC
34839	A	G	psbC
35002	C	T	psbC
35016	C	T	psbC
35046	C	T	psbC
35091	T	C	psbC
35148	C	T	psbC
35350	T	G	psbC-psbZ
35402	C	T	psbC-psbZ
35409	C	T	psbC-psbZ
35422	T	G	psbC-psbZ
35485	G	A	psbC-psbZ
35495	C	T	psbC-psbZ
35522	A	C	psbC-psbZ
35690	G	A	psbC-psbZ
35727	A	C	psbC-psbZ
35759	A	G	psbC-psbZ
35804	C	T	psbC-psbZ
35813	G	T	psbC-psbZ
35818	A	C	psbC-psbZ
35820	A	T	psbC-psbZ
35829	T	C	psbC-psbZ
35829	T	G	psbC-psbZ
35901	T	C	psbC-psbZ
35936	C	T	psbC-psbZ
35971	G	A	psbC-psbZ
36010	C	T	psbZ
36049	C	A	psbZ
36071	A	G	psbZ
36258	A	C	psbZ-rps14
36287	G	A	psbZ-rps14
36360	G	A	psbZ-rps14
36373	A	C	psbZ-rps14
36434	A	C	psbZ-rps14
36468	C	T	psbZ-rps14
36551	G	A	psbZ-rps14
36571	A	C	psbZ-rps14
36828	T	C	psbZ-rps14
36853	G	A	psbZ-rps14
36860	T	C	psbZ-rps14

36957	G	A	rps14
36959	T	G	rps14
37078	C	T	rps14
37260	A	C	rps14-psaB
37263	T	G	rps14-psaB
37264	A	C	rps14-psaB
37271	T	G	rps14-psaB
37758	A	G	psaB
37779	A	G	psaB
37809	C	T	psaB
37947	C	T	psaB
38052	G	A	psaB
38058	C	A	psaB
38551	C	T	psaB
38568	C	G	psaB
38706	C	T	psaB
38760	G	A	psaB
39045	T	C	psaB
39069	A	C	psaB
39083	A	G	psaB
39192	A	G	psaB
39198	C	T	psaB
39201	G	A	psaB
39817	G	A	psaA
39820	G	T	psaA
39898	A	G	psaA
40051	A	C	psaA
40060	A	G	psaA
40134	G	A	psaA
40315	G	A	psaA
40587	G	A	psaA
40663	T	C	psaA
41027	T	A	psaA
41101	A	G	psaA
41222	G	T	psaA
41332	T	C	psaA
41362	A	G	psaA
41407	C	T	psaA
41584	T	C	psaA
41662	G	A	psaA
41670	C	T	psaA
41751	C	T	psaA
41763	A	G	psaA
41837	G	T	psaA-ycf3

41896	G	T	psaA-ycf3
41908	C	T	psaA-ycf3
42004	C	T	psaA-ycf3
42021	T	C	psaA-ycf3
42029	A	G	psaA-ycf3
42091	G	A	psaA-ycf3
42169	A	G	psaA-ycf3
42192	T	C	psaA-ycf3
42207	A	C	psaA-ycf3
42257	A	C	psaA-ycf3
42262	A	G	psaA-ycf3
42283	C	T	psaA-ycf3
42408	C	T	ycf3
42484	T	C	ycf3
42528	C	T	ycf3
42559	A	C	ycf3
42560	T	C	ycf3
42723	A	G	ycf3
42739	T	A	ycf3
42742	A	T	ycf3
42752	A	C	ycf3
42764	C	T	ycf3
42892	T	C	ycf3
42897	G	A	ycf3
42926	G	A	ycf3
42929	A	C	ycf3
42960	G	A	ycf3
42977	T	G	ycf3
42994	C	T	ycf3
43015	T	G	ycf3
43050	C	T	ycf3
43218	A	C	ycf3
43226	C	T	ycf3
43307	T	C	ycf3
43434	C	T	ycf3
43454	C	T	ycf3
43508	T	C	ycf3
43616	T	G	ycf3
43741	T	C	ycf3
43811	A	G	ycf3
43998	A	C	ycf3
44032	C	T	ycf3
44045	A	G	ycf3
44200	T	C	ycf3

44407	A	C	ycf3-rps4
44500	A	G	ycf3-rps4
44508	T	G	ycf3-rps4
44596	A	G	ycf3-rps4
44689	T	G	ycf3-rps4
44694	A	G	ycf3-rps4
44711	T	C	ycf3-rps4
44814	G	T	ycf3-rps4
44826	A	G	ycf3-rps4
44837	G	A	ycf3-rps4
44887	A	G	ycf3-rps4
44940	G	T	ycf3-rps4
44948	T	C	ycf3-rps4
45056	C	A	ycf3-rps4
45069	A	C	ycf3-rps4
45150	G	A	ycf3-rps4
45158	C	T	ycf3-rps4
45194	C	T	ycf3-rps4
45270	C	T	ycf3-rps4
45318	G	T	ycf3-rps4
45319	A	C	ycf3-rps4
45380	G	T	ycf3-rps4
45440	G	A	ycf3-rps4
45466	C	T	ycf3-rps4
45610	T	C	rps4
45752	C	T	rps4
46047	C	T	rps4
46158	C	A	rps4
46227	A	T	rps4-ndhJ
46227	A	C	rps4-ndhJ
46237	T	G	rps4-ndhJ
46315	A	T	rps4-ndhJ
46357	C	T	rps4-ndhJ
46372	T	C	rps4-ndhJ
46373	T	G	rps4-ndhJ
46410	T	G	rps4-ndhJ
46429	G	A	rps4-ndhJ
46432	G	A	rps4-ndhJ
46588	A	G	rps4-ndhJ
46593	C	T	rps4-ndhJ
46599	G	A	rps4-ndhJ
46640	A	G	rps4-ndhJ
46664	A	T	rps4-ndhJ
46665	A	C	rps4-ndhJ

46773	G	T	rps4-ndhJ
46891	C	T	rps4-ndhJ
46906	T	G	rps4-ndhJ
46911	T	G	rps4-ndhJ
46917	T	G	rps4-ndhJ
46922	C	A	rps4-ndhJ
46927	A	C	rps4-ndhJ
46993	T	G	rps4-ndhJ
47060	T	G	rps4-ndhJ
47078	A	G	rps4-ndhJ
47086	G	A	rps4-ndhJ
47129	A	G	rps4-ndhJ
47189	G	A	rps4-ndhJ
47203	C	T	rps4-ndhJ
47248	A	C	rps4-ndhJ
47279	C	A	rps4-ndhJ
47312	C	T	rps4-ndhJ
47428	A	G	rps4-ndhJ
47482	T	G	rps4-ndhJ
47526	A	G	rps4-ndhJ
47541	C	A	rps4-ndhJ
47551	A	G	rps4-ndhJ
47607	C	T	rps4-ndhJ
47638	G	A	rps4-ndhJ
47668	C	G	rps4-ndhJ
47707	A	C	rps4-ndhJ
47811	T	C	rps4-ndhJ
47921	G	A	rps4-ndhJ
47999	C	T	rps4-ndhJ
48052	C	T	rps4-ndhJ
48125	G	T	rps4-ndhJ
48137	G	T	rps4-ndhJ
48224	C	T	rps4-ndhJ
48225	G	A	rps4-ndhJ
48311	T	G	rps4-ndhJ
48357	G	A	rps4-ndhJ
48458	T	G	rps4-ndhJ
48463	A	C	rps4-ndhJ
48636	T	C	rps4-ndhJ
48764	G	T	rps4-ndhJ
48848	C	T	rps4-ndhJ
48882	A	G	rps4-ndhJ
48913	T	C	rps4-ndhJ
48947	T	G	rps4-ndhJ

48954	C	A	rps4-ndhJ
48965	G	T	rps4-ndhJ
48994	C	T	rps4-ndhJ
49036	T	G	rps4-ndhJ
49044	C	T	rps4-ndhJ
49045	C	T	rps4-ndhJ
49071	T	G	rps4-ndhJ
49072	C	A	rps4-ndhJ
49342	T	G	ndhJ
49465	C	T	ndhJ
49549	C	A	ndhJ
49608	T	G	ndhJ-matK
49615	T	G	ndhJ-matK
49676	C	T	ndhK
49697	A	G	ndhK
49741	G	T	ndhK
49767	G	T	ndhK
49877	G	A	ndhK
49884	C	T	ndhK
50003	A	C	ndhK
50060	T	C	ndhK
50078	T	C	ndhK
50117	T	G	ndhK
50156	A	G	ndhK
50593	T	G	ndhC
50612	G	A	ndhC
50696	T	G	ndhC
50758	G	A	ndhC
50769	G	A	ndhC-atpE
50803	C	G	ndhC-atpE
50812	T	C	ndhC-atpE
50823	C	T	ndhC-atpE
50876	A	G	ndhC-atpE
50902	G	A	ndhC-atpE
50925	C	T	ndhC-atpE
50959	G	T	ndhC-atpE
51017	G	A	ndhC-atpE
51041	A	T	ndhC-atpE
51043	T	G	ndhC-atpE
51054	G	T	ndhC-atpE
51082	A	C	ndhC-atpE
51088	C	A	ndhC-atpE
51111	T	G	ndhC-atpE
51117	G	A	ndhC-atpE

51123	T	G	ndhC-atpE
51150	T	G	ndhC-atpE
51165	G	A	ndhC-atpE
51206	G	A	ndhC-atpE
51214	G	A	ndhC-atpE
51235	G	T	ndhC-atpE
51249	A	C	ndhC-atpE
51292	T	C	ndhC-atpE
51297	G	A	ndhC-atpE
51503	T	C	ndhC-atpE
51575	A	C	ndhC-atpE
51597	C	A	ndhC-atpE
51625	T	G	ndhC-atpE
51627	A	C	ndhC-atpE
51628	G	A	ndhC-atpE
51660	G	T	ndhC-atpE
51678	A	G	ndhC-atpE
51730	G	T	ndhC-atpE
51794	A	T	ndhC-atpE
51794	A	C	ndhC-atpE
51815	T	C	ndhC-atpE
51863	A	C	ndhC-atpE
51929	C	T	ndhC-atpE
52044	T	G	ndhC-atpE
52062	T	G	ndhC-atpE
52091	A	G	ndhC-atpE
52274	T	G	ndhC-atpE
52346	G	A	ndhC-atpE
52472	C	A	ndhC-atpE
52481	G	A	ndhC-atpE
52543	C	T	ndhC-atpE
52593	T	C	ndhC-atpE
52615	C	T	ndhC-atpE
52621	C	T	ndhC-atpE
52708	C	G	ndhC-atpE
52799	T	C	ndhC-atpE
53011	C	T	atpE
53084	C	A	atpE
53168	G	T	atpE
53246	C	A	atpE
53815	A	C	atpB
53827	C	T	atpB
53836	T	C	atpB
54289	G	T	atpB

54586	A	G	atpB
54603	G	A	atpB
54700	C	T	atpB
54703	C	T	atpB
54750	C	T	atpB
54952	T	G	atpB-rbcL
54970	A	G	atpB-rbcL
55003	A	G	atpB-rbcL
55066	A	G	atpB-rbcL
55122	A	C	atpB-rbcL
55244	T	G	atpB-rbcL
55267	A	G	atpB-rbcL
55275	C	T	atpB-rbcL
55344	A	C	atpB-rbcL
55400	A	C	atpB-rbcL
55407	A	C	atpB-rbcL
55663	G	A	rbcL
55719	C	G	rbcL
55806	T	A	rbcL
55941	T	C	rbcL
55950	T	C	rbcL
56017	T	C	rbcL
56024	C	T	rbcL
56164	T	C	rbcL
56189	C	A	rbcL
56245	T	C	rbcL
56267	A	C	rbcL
56299	G	A	rbcL
56498	T	G	rbcL
56518	A	G	rbcL
56653	A	G	rbcL
56761	G	A	rbcL
56782	A	C	rbcL
56844	A	C	rbcL
57069	T	G	rbcL-psaI
57137	C	T	rbcL-psaI
57139	C	A	rbcL-psaI
57248	T	C	rbcL-psaI
57258	C	T	rbcL-psaI
57281	C	T	rbcL-psaI
57331	T	G	rbcL-psaI
57413	C	T	rbcL-psaI
57431	G	T	rbcL-psaI
57438	A	C	rbcL-psaI

57463	G	C	rbcL-psaI
57503	A	C	rbcL-psaI
57508	T	G	rbcL-psaI
57512	C	T	rbcL-psaI
57559	T	C	rbcL-psaI
57567	C	A	rbcL-psaI
57647	A	G	rbcL-psaI
57658	C	T	rbcL-psaI
57679	G	A	rbcL-psaI
57687	G	A	rbcL-psaI
57688	G	A	rbcL-psaI
57731	G	A	rbcL-psaI
57766	T	G	rbcL-psaI
57811	A	C	rbcL-psaI
57924	G	A	rbcL-psaI
57974	C	T	rbcL-psaI
58118	C	A	rbcL-psaI
58148	A	G	rbcL-psaI
58179	A	C	rbcL-psaI
58197	T	G	rbcL-psaI
58207	G	A	rbcL-psaI
58292	T	G	rbcL-psaI
58293	G	A	rbcL-psaI
58360	G	A	rbcL-psaI
58368	T	C	rbcL-psaI
58433	G	A	rbcL-psaI
58448	G	A	rbcL-psaI
58470	A	G	rbcL-psaI
58472	T	C	rbcL-psaI
58506	C	T	rbcL-psaI
58530	A	T	rbcL-psaI
58550	A	T	rbcL-psaI
58593	G	T	rbcL-psaI
58626	T	G	rbcL-psaI
58633	C	A	rbcL-psaI
58648	A	G	rbcL-psaI
58699	G	A	rbcL-psaI
58729	A	G	rbcL-psaI
58784	C	T	rbcL-psaI
58791	G	A	rbcL-psaI
58964	C	T	rbcL-psaI
58967	G	A	rbcL-psaI
58985	C	T	rbcL-psaI
58987	C	T	rbcL-psaI

59013	G	A	rbcL-psaI
59114	A	G	rbcL-psaI
59147	G	T	rbcL-psaI
59172	A	C	rbcL-psaI
59195	A	C	rbcL-psaI
59210	T	C	rbcL-psaI
59228	C	A	rbcL-psaI
59231	G	A	rbcL-psaI
59315	A	C	rbcL-psaI
59402	A	C	rbcL-psaI
59430	C	A	rbcL-psaI
59455	C	T	rbcL-psaI
59519	C	A	rbcL-psaI
59545	G	A	rbcL-psaI
59551	G	T	rbcL-psaI
59603	T	G	rbcL-psaI
59624	A	T	rbcL-psaI
59630	A	C	rbcL-psaI
59705	T	C	rbcL-psaI
59771	A	G	psaI
59816	G	A	psaI
60203	C	A	ycf4
60221	A	C	ycf4
60308	G	T	ycf4
60468	T	C	ycf4
60559	A	G	ycf4
60664	T	G	ycf4
60688	C	T	ycf4
60698	C	A	ycf4
60712	A	C	ycf4
60747	G	A	ycf4-cemA
60763	A	C	ycf4-cemA
60766	G	A	ycf4-cemA
60869	A	C	ycf4-cemA
60874	T	G	ycf4-cemA
60932	C	T	ycf4-cemA
60949	T	G	ycf4-cemA
60971	T	G	ycf4-cemA
61029	G	T	ycf4-cemA
61077	T	C	ycf4-cemA
61173	A	C	ycf4-cemA
61196	A	G	ycf4-cemA
61210	A	C	ycf4-cemA
61223	G	T	ycf4-cemA

61242	G	A	cemA
61283	G	T	cemA
61406	A	C	cemA
61421	C	T	cemA
61520	C	T	cemA
61838	T	G	cemA
61961	G	A	cemA-petA
61977	G	T	cemA-petA
62021	G	A	cemA-petA
62058	T	G	cemA-petA
62079	C	T	cemA-petA
62104	A	C	cemA-petA
62111	C	T	cemA-petA
62141	G	A	cemA-petA
62223	A	C	petA
62314	G	A	petA
62326	C	T	petA
62344	C	T	petA
62354	G	A	petA
62530	T	G	petA
62554	G	A	petA
62632	C	A	petA
62692	C	T	petA
62915	A	C	petA
62922	T	G	petA
62927	C	G	petA
62975	G	A	petA
63001	A	C	petA
63121	G	A	petA-psbJ
63150	A	G	petA-psbJ
63165	G	A	petA-psbJ
63167	G	A	petA-psbJ
63181	C	A	petA-psbJ
63191	T	G	petA-psbJ
63200	A	C	petA-psbJ
63200	A	T	petA-psbJ
63256	C	T	petA-psbJ
63301	C	A	petA-psbJ
63321	T	A	petA-psbJ
63355	T	G	petA-psbJ
63400	A	C	petA-psbJ
63404	C	T	petA-psbJ
63523	C	T	petA-psbJ
63547	C	A	petA-psbJ

63554	C	T	petA-psbJ
63578	G	A	petA-psbJ
63606	C	T	petA-psbJ
63671	T	C	petA-psbJ
63699	C	T	petA-psbJ
63741	T	C	petA-psbJ
63770	T	G	petA-psbJ
63781	C	A	petA-psbJ
63838	A	C	petA-psbJ
63857	T	C	petA-psbJ
63863	T	C	petA-psbJ
63891	A	C	petA-psbJ
63948	C	T	petA-psbJ
63988	T	G	petA-psbJ
63989	G	A	petA-psbJ
64029	G	T	petA-psbJ
64051	T	C	petA-psbJ
64094	C	A	petA-psbJ
64266	T	C	psbJ-psbL
64323	A	G	psbJ-psbL
64327	T	C	psbJ-psbL
64575	G	A	psbF
64646	G	C	psbF
64739	C	T	psbE
64778	A	G	psbE
64981	G	T	psbE-petL
64982	A	C	psbE-petL
65092	C	T	psbE-petL
65134	C	T	psbE-petL
65151	A	G	psbE-petL
65163	T	G	psbE-petL
65168	C	T	psbE-petL
65192	G	T	psbE-petL
65198	G	T	psbE-petL
65208	G	T	psbE-petL
65231	T	G	psbE-petL
65240	G	A	psbE-petL
65251	G	T	psbE-petL
65259	C	T	psbE-petL
65292	C	A	psbE-petL
65295	T	C	psbE-petL
65314	A	G	psbE-petL
65343	A	G	psbE-petL
65346	C	A	psbE-petL

65347	C	A	psbE-petL
65366	A	C	psbE-petL
65372	G	C	psbE-petL
65373	T	G	psbE-petL
65374	C	A	psbE-petL
65430	A	C	psbE-petL
65463	G	A	psbE-petL
65502	G	T	psbE-petL
65513	A	C	psbE-petL
65548	G	C	psbE-petL
65570	A	C	psbE-petL
65616	A	C	psbE-petL
65626	C	A	psbE-petL
65663	A	C	psbE-petL
65678	G	T	psbE-petL
65679	A	G	psbE-petL
65679	A	T	psbE-petL
65682	C	T	psbE-petL
65706	A	C	psbE-petL
65712	A	C	psbE-petL
65718	A	G	psbE-petL
65770	G	A	psbE-petL
65792	C	T	psbE-petL
65855	A	C	psbE-petL
65860	G	A	psbE-petL
65865	A	C	psbE-petL
65890	C	T	psbE-petL
65935	A	G	psbE-petL
65969	A	C	psbE-petL
65975	A	C	psbE-petL
66090	T	G	petL-petG
66138	G	A	petL-petG
66175	A	C	petL-petG
66343	G	A	petG-psaJ
66370	C	T	petG-psaJ
66386	G	A	petG-psaJ
66571	T	G	petG-psaJ
66575	G	A	petG-psaJ
66619	A	C	petG-psaJ
66625	A	C	petG-psaJ
66635	T	G	petG-psaJ
66668	C	G	petG-psaJ
66861	A	T	petG-psaJ
66905	C	G	petG-psaJ

66945	T	G	petG-psaJ
66956	A	C	petG-psaJ
66963	G	A	petG-psaJ
66995	A	G	petG-psaJ
67006	A	G	petG-psaJ
67073	C	G	petG-psaJ
67096	C	T	petG-psaJ
67176	A	C	petG-psaJ
67306	A	G	psaJ
67318	G	T	psaJ
67375	A	C	psaJ-rpl33
67485	T	G	psaJ-rpl33
67489	A	T	psaJ-rpl33
67549	T	G	psaJ-rpl33
67605	C	T	psaJ-rpl33
67635	A	C	psaJ-rpl33
67638	T	G	psaJ-rpl33
67693	T	C	psaJ-rpl33
67833	T	C	rpl33
67840	A	C	rpl33
67895	A	G	rpl33
67949	C	T	rpl33
67951	A	G	rpl33
68026	T	C	rpl33-rps18
68084	A	C	rpl33-rps18
68164	A	G	rpl33-rps18
68205	C	A	rps18
68255	T	C	rps18
68450	T	C	rps18
68452	A	G	rps18
68464	G	C	rps18
68533	T	C	rps18-rpl20
68559	G	A	rps18-rpl20
68571	G	A	rps18-rpl20
68592	G	A	rps18-rpl20
68607	G	A	rps18-rpl20
68692	C	A	rps18-rpl20
68714	G	T	rps18-rpl20
68734	C	T	rps18-rpl20
68775	G	T	rps18-rpl20
68922	G	A	rpl20
68968	A	C	rpl20
69063	G	A	rpl20
69107	C	T	rpl20-rps12

69151	T	C	rpl20-rps12
69248	A	C	rpl20-rps12
69309	T	G	rpl20-rps12
69314	T	G	rpl20-rps12
69368	C	A	rpl20-rps12
69411	G	A	rpl20-rps12
69447	A	C	rpl20-rps12
69454	A	C	rpl20-rps12
69547	C	T	rpl20-rps12
69588	A	G	rpl20-rps12
69608	T	C	rpl20-rps12
69620	G	A	rpl20-rps12
69699	G	T	rpl20-rps12
69704	T	G	rpl20-rps12
69767	G	T	rpl20-rps12
69776	A	C	rpl20-rps12
69810	T	G	rpl20-rps12
69825	T	C	rpl20-rps12
69853	A	T	rpl20-rps12
69874	C	T	rpl20-rps12
70066	A	C	rps12-clpP
70081	T	A	rps12-clpP
70086	A	T	rps12-clpP
70100	G	T	rps12-clpP
70106	C	T	rps12-clpP
70133	C	T	rps12-clpP
70268	A	G	clpP
70307	C	T	clpP
70370	G	A	clpP
70409	T	G	clpP
70441	G	C	clpP
70441	G	A	clpP
70472	A	C	clpP
70544	A	C	clpP
70629	T	G	clpP
70648	C	T	clpP
70650	G	A	clpP
70674	C	T	clpP
70736	C	T	clpP
70753	T	G	clpP
70762	A	C	clpP
70795	G	T	clpP
70805	C	T	clpP
70853	A	C	clpP

70857	G	T	clpP
70901	T	G	clpP
70925	T	G	clpP
70968	A	G	clpP
71027	T	G	clpP
71108	T	C	clpP
71152	T	G	clpP
71276	T	C	clpP
71403	G	A	clpP
71491	G	A	clpP
71529	C	T	clpP
71556	G	A	clpP
71578	C	A	clpP
71583	A	C	clpP
71628	T	G	clpP
71740	C	T	clpP
71763	A	C	clpP
71769	G	T	clpP
71770	A	C	clpP
71776	C	A	clpP
71791	T	C	clpP
71829	T	C	clpP
71842	G	T	clpP
71845	C	T	clpP
71943	A	C	clpP
72001	T	G	clpP
72017	A	G	clpP
72045	C	T	clpP
72056	T	G	clpP
72070	A	C	clpP
72120	T	C	clpP
72124	C	T	clpP
72397	A	G	clpP-psbB
72542	G	C	clpP-psbB
72587	C	T	clpP-psbB
72645	G	T	clpP-psbB
72679	C	T	clpP-psbB
72935	C	T	psbB
73274	T	C	psbB
73367	C	T	psbB
73370	G	A	psbB
73436	T	C	psbB
73601	A	C	psbB
73608	G	A	psbB

73652	A	C	psbB
73733	G	A	psbB
73757	A	C	psbB
74012	G	T	psbB
74231	C	T	psbB
74245	T	C	psbB-psbT
74366	C	A	psbB-psbT
74596	T	C	psbN
74599	G	A	psbN
74819	G	A	psbN-psbH
74850	T	C	psbH
74919	G	A	psbH
75051	G	T	psbH
75066	A	G	psbH-petB
75281	G	A	petB
75317	G	A	petB
75393	T	A	petB
75406	G	T	petB
75419	A	G	petB
75465	T	G	petB
75504	G	A	petB
75510	A	G	petB
75653	T	G	petB
75697	C	T	petB
75709	T	G	petB
75887	G	A	petB
75899	T	G	petB
76361	A	G	petB
76662	A	C	petB-petD
76725	A	C	petB-petD
76744	C	T	petB-petD
76845	C	T	petD-rpoA
76847	G	A	petD-rpoA
76930	A	G	petD-rpoA
77075	G	A	petD-rpoA
77086	A	C	petD-rpoA
77159	C	G	petD-rpoA
77245	C	T	petD-rpoA
77320	T	C	petD-rpoA
77350	A	C	petD-rpoA
77379	C	A	petD-rpoA
77395	T	C	petD-rpoA
77396	G	T	petD-rpoA
77477	C	T	petD-rpoA

77613	T	C	petD-rpoA
77721	A	C	petD-rpoA
77907	T	C	petD-rpoA
77970	T	C	petD-rpoA
77986	G	A	petD-rpoA
78009	G	A	petD-rpoA
78030	C	T	petD-rpoA
78078	G	T	petD-rpoA
78080	A	C	petD-rpoA
78093	A	G	petD-rpoA
78114	G	T	petD-rpoA
78279	A	T	petD-rpoA
78285	A	C	rpoA
78477	T	G	rpoA
78567	G	C	rpoA
78567	G	A	rpoA
78625	T	C	rpoA
78639	A	G	rpoA
78762	C	T	rpoA
78965	G	A	rpoA
78990	T	G	rpoA
79008	C	T	rpoA
79097	A	G	rpoA
79131	C	T	rpoA
79132	T	C	rpoA
79135	T	G	rpoA
79153	G	A	rpoA
79158	T	C	rpoA
79209	G	A	rpoA
79336	C	A	rpoA-rps11
79358	T	C	rps11
79366	T	G	rps11
79367	A	G	rps11
79400	G	T	rps11
79550	G	A	rps11
79717	C	T	rps11
79720	C	T	rps11
79734	T	C	rps11
79736	C	T	rps11
79749	G	A	rps11
79836	C	T	rps11-rpl36
79837	A	G	rps11-rpl36
79908	T	C	rpl36
79968	G	T	rpl36

80012	G	T	rpl36-rps8
80024	G	A	rpl36-rps8
80041	G	T	rpl36-rps8
80042	A	C	rpl36-rps8
80071	G	A	rpl36-rps8
80106	T	C	rpl36-rps8
80132	G	T	rpl36-rps8
80209	C	T	rpl36-rps8
80232	A	C	rpl36-rps8
80235	G	T	rpl36-rps8
80274	C	T	rpl36-rps8
80393	A	C	rpl36-rps8
80398	C	A	rpl36-rps8
80410	G	A	rpl36-rps8
80419	C	A	rpl36-rps8
80445	T	C	rpl36-rps8
80503	T	C	rps8
80537	G	T	rps8
80547	C	G	rps8
80586	A	C	rps8
80598	T	C	rps8
80642	G	A	rps8
80653	C	A	rps8
80668	C	T	rps8
80811	C	T	rps8
80847	A	G	rps8
80918	A	T	rps8-rpl14
80943	G	T	rps8-rpl14
80945	T	G	rps8-rpl14
80951	T	G	rps8-rpl14
81011	T	C	rps8-rpl14
81456	G	A	rpl14-rpl16
81576	C	T	rpl16
81599	T	C	rpl16
81621	T	C	rpl16
81930	G	C	rpl16
82033	G	A	rpl16
82083	A	C	rpl16
82115	G	T	rpl16
82161	T	C	rpl16
82172	T	G	rpl16
82173	A	C	rpl16
82200	C	T	rpl16
82206	A	C	rpl16

82268	G	A	rpl16
82322	T	G	rpl16
82335	C	T	rpl16
82352	A	T	rpl16
82356	G	T	rpl16
82445	T	C	rpl16
82458	G	T	rpl16
82475	C	A	rpl16
82570	C	A	rpl16
82637	G	T	rpl16
82681	C	A	rpl16
82689	T	C	rpl16
82728	G	T	rpl16
82751	T	A	rpl16
82766	C	A	rpl16
82803	T	G	rpl16
82812	G	T	rpl16
82813	A	C	rpl16
82828	C	T	rpl16
82893	C	T	rpl16
82966	T	A	rpl16-rps3
83008	A	C	rpl16-rps3
83009	A	G	rpl16-rps3
83039	A	G	rpl16-rps3
83135	C	T	rps3
83190	G	A	rps3
83321	C	T	rps3
83531	T	G	rps3
83688	C	T	rps3
83719	A	C	rps3
83747	C	T	rps3
83797	C	T	rpl22
83970	C	T	rpl22
83996	G	T	rpl22
84132	C	T	rpl22
84245	C	T	rpl22-rps19
84344	T	C	rps19
84361	T	A	
84488	C	A	rps19
84624	A	C	rpl2
109549	A	G	ndhF
109672	G	A	ndhF
109687	G	A	ndhF
109731	T	C	ndhF

109737	C	G	ndhF
109762	C	T	ndhF
109820	G	T	ndhF
109861	G	A	ndhF
109962	T	G	ndhF
109986	T	G	ndhF
109997	T	G	ndhF
110078	A	G	ndhF
110113	A	G	ndhF
110199	T	C	ndhF
110283	T	C	ndhF
110301	C	T	ndhF
110580	G	C	ndhF
110591	A	G	ndhF
110625	C	T	ndhF
110644	T	C	ndhF
110751	C	G	ndhF
110807	G	A	ndhF
110814	G	A	ndhF
110966	T	C	ndhF
110979	T	C	ndhF
111113	G	T	ndhF
111152	T	C	ndhF
111178	A	T	ndhF
111292	C	A	ndhF-rpl32
111304	T	G	ndhF-rpl32
111308	C	T	ndhF-rpl32
111329	G	A	ndhF-rpl32
111330	G	A	ndhF-rpl32
111372	T	G	ndhF-rpl32
111429	T	C	ndhF-rpl32
111505	T	G	ndhF-rpl32
111507	G	A	ndhF-rpl32
111515	C	T	ndhF-rpl32
111552	C	A	ndhF-rpl32
111610	T	G	ndhF-rpl32
111618	A	T	ndhF-rpl32
111620	A	T	ndhF-rpl32
111621	T	G	ndhF-rpl32
111642	A	G	ndhF-rpl32
111670	A	T	ndhF-rpl32
111681	T	C	ndhF-rpl32
111711	A	T	ndhF-rpl32
111721	A	T	ndhF-rpl32

111729	T	C	ndhF-rpl32
111739	T	A	ndhF-rpl32
111740	T	A	ndhF-rpl32
111744	T	A	ndhF-rpl32
111746	G	A	ndhF-rpl32
111750	C	T	ndhF-rpl32
111756	C	T	ndhF-rpl32
111757	A	C	ndhF-rpl32
111778	G	T	ndhF-rpl32
111783	C	A	ndhF-rpl32
111784	T	A	ndhF-rpl32
111786	C	A	ndhF-rpl32
111816	C	A	ndhF-rpl32
111834	T	G	ndhF-rpl32
111835	C	A	ndhF-rpl32
111851	A	T	ndhF-rpl32
111856	T	C	ndhF-rpl32
111892	G	T	ndhF-rpl32
111900	C	T	ndhF-rpl32
111901	A	G	ndhF-rpl32
111917	T	G	ndhF-rpl32
111921	T	G	ndhF-rpl32
111922	C	T	ndhF-rpl32
111941	G	A	ndhF-rpl32
111942	C	T	ndhF-rpl32
111997	G	A	ndhF-rpl32
112086	C	T	rpl32
112087	A	G	rpl32
112211	C	A	rpl32-ccsA
112219	G	T	rpl32-ccsA
112239	C	G	rpl32-ccsA
112250	C	T	rpl32-ccsA
112306	A	C	rpl32-ccsA
112369	T	G	rpl32-ccsA
112389	G	T	rpl32-ccsA
112451	T	G	rpl32-ccsA
112524	C	T	rpl32-ccsA
112546	A	G	rpl32-ccsA
112559	T	G	rpl32-ccsA
112576	T	A	rpl32-ccsA
112598	T	G	rpl32-ccsA
112599	A	C	rpl32-ccsA
112745	T	G	rpl32-ccsA
112759	T	G	rpl32-ccsA

112765	A	G	rpl32-ccsA
112766	G	A	rpl32-ccsA
112782	C	A	rpl32-ccsA
112816	T	C	rpl32-ccsA
112829	A	T	rpl32-ccsA
112876	A	C	ccsA
112922	C	T	ccsA
112932	C	T	ccsA
112976	T	C	ccsA
113112	A	T	ccsA
113184	G	A	ccsA
113295	C	T	ccsA
113316	A	G	ccsA
113350	C	T	ccsA
113361	T	C	ccsA
113372	G	T	ccsA
113385	T	G	ccsA
113391	T	A	ccsA
113414	C	A	ccsA
113441	G	A	ccsA
113450	A	C	ccsA
113462	T	C	ccsA
113675	C	A	ccsA
113677	A	C	ccsA
113692	G	A	ccsA
113769	T	C	ccsA
113928	A	G	ccsA-ndhD
113950	A	C	ccsA-ndhD
113973	T	G	ccsA-ndhD
113975	G	C	ccsA-ndhD
113976	G	C	ccsA-ndhD
114018	C	A	ccsA-ndhD
114047	T	G	ndhD
114105	C	G	ndhD
114147	T	G	ndhD
114151	G	A	ndhD
114175	T	C	ndhD
114191	G	T	ndhD
114193	A	C	ndhD
114239	A	G	ndhD
114279	T	C	ndhD
114300	A	G	ndhD
114321	A	G	ndhD
114322	A	T	ndhD

114323	T	A	ndhD
114337	T	G	ndhD
114498	C	T	ndhD
114573	T	C	ndhD
114588	T	C	ndhD
114628	A	G	ndhD
114718	A	C	ndhD
114843	C	T	ndhD
114964	T	G	ndhD
115042	C	T	ndhD
115072	T	C	ndhD
115081	G	C	ndhD
115177	A	G	ndhD
115249	C	A	ndhD
115326	C	G	ndhD
115326	C	T	ndhD
115384	G	A	ndhD
115405	G	A	ndhD
115457	A	G	ndhD
115483	G	A	ndhD
115517	G	A	ndhD
115570	G	A	ndhD-psaC
115629	G	A	ndhD-psaC
115758	A	G	psaC
115937	A	G	psaC-ndhE
115948	T	G	psaC-ndhE
115987	C	A	psaC-ndhE
115995	A	C	psaC-ndhE
116002	A	G	psaC-ndhE
116031	A	C	psaC-ndhE
116037	G	T	psaC-ndhE
116067	T	G	psaC-ndhE
116116	T	C	psaC-ndhE
116139	C	T	psaC-ndhE
116161	T	G	psaC-ndhE
116162	A	C	psaC-ndhE
116180	A	C	psaC-ndhE
116361	A	C	ndhE
116481	G	T	ndhE
116573	G	A	ndhE-ndhG
116597	T	G	ndhE-ndhG
116603	T	C	ndhE-ndhG
116624	C	T	ndhE-ndhG
116638	G	A	ndhE-ndhG

116656	T	C	ndhE-ndhG
116675	T	C	ndhE-ndhG
116920	C	T	ndhG
116958	C	T	ndhG
116967	A	C	ndhG
116996	C	T	ndhG
117014	G	A	ndhG
117080	T	C	ndhG
117168	G	A	ndhG
117199	C	A	ndhG
117289	A	G	ndhG-ndhI
117360	G	A	ndhG-ndhI
117369	G	T	ndhG-ndhI
117370	G	A	ndhG-ndhI
117383	C	A	ndhG-ndhI
117483	T	G	ndhG-ndhI
117488	G	A	ndhG-ndhI
117510	C	T	ndhG-ndhI
117520	T	G	ndhG-ndhI
117607	T	G	ndhI
117636	A	C	ndhI
117637	T	C	ndhI
117739	A	G	ndhI
117841	A	G	ndhI
117850	G	T	ndhI
117881	G	A	ndhI
117990	G	A	ndhI
118027	T	C	ndhI
118150	G	T	ndhI-ndhA
118154	C	T	ndhI-ndhA
118166	T	C	ndhA
118185	G	A	ndhA
118261	A	G	ndhA
118363	C	T	ndhA
118368	G	T	ndhA
118424	T	C	ndhA
118705	G	A	ndhA
118745	C	T	ndhA
118816	G	T	ndhA
118846	T	C	ndhA
118965	A	C	ndhA
119085	G	C	ndhA
119095	C	T	ndhA
119109	T	G	ndhA

119250	T	C	ndhA
119307	C	T	ndhA
119341	G	A	ndhA
119362	G	T	ndhA
119415	A	G	ndhA
119457	T	C	ndhA
119498	C	A	ndhA
119588	C	T	ndhA
119596	T	G	ndhA
119615	C	A	ndhA
119623	C	T	ndhA
119697	A	C	ndhA
119723	G	A	ndhA
119727	C	G	ndhA
119728	A	C	ndhA
119739	T	C	ndhA
119747	C	T	ndhA
119771	T	G	ndhA
119779	C	A	ndhA
119880	G	A	ndhA
120003	A	G	ndhA
120065	G	T	ndhA
120189	T	A	ndhA
120222	A	G	ndhA
120469	C	T	ndhH
120489	G	T	ndhH
120610	G	A	ndhH
120648	C	T	ndhH
120650	C	T	ndhH
120718	G	T	ndhH
120802	G	A	ndhH
120813	T	C	ndhH
120853	G	T	ndhH
120893	T	C	ndhH
120980	G	A	ndhH
121021	A	G	ndhH
121026	T	C	ndhH
121147	A	G	ndhH
121156	C	T	ndhH
121171	T	C	ndhH
121183	T	C	ndhH
121306	T	G	ndhH
121336	T	C	ndhH
121369	A	G	ndhH

121425	C	T	ndhH
121509	C	T	ndhH
121530	G	A	ndhH
121531	T	C	ndhH
121541	G	A	ndhH-rps15
121561	T	C	ndhH-rps15
121580	A	C	ndhH-rps15
121597	A	C	ndhH-rps15
121607	G	T	ndhH-rps15
121607	G	A	ndhH-rps15
121608	A	C	ndhH-rps15
121733	C	A	rps15
121879	A	C	rps15
121896	T	G	rps15-ycf1
121929	G	A	rps15-ycf1
121951	T	G	rps15-ycf1
121987	A	T	rps15-ycf1
122000	G	T	rps15-ycf1
122001	A	C	rps15-ycf1
122053	T	G	rps15-ycf1
122083	C	A	rps15-ycf1
122088	T	G	rps15-ycf1
122189	A	C	rps15-ycf1
122194	G	A	rps15-ycf1
122259	C	A	rps15-ycf1
122394	T	G	ycf1
122403	T	G	ycf1
122449	G	T	ycf1
122457	G	T	ycf1
122482	C	A	ycf1
122494	A	G	ycf1
122527	C	A	ycf1
122538	T	C	ycf1
122574	A	G	ycf1
122590	T	C	ycf1
122607	C	A	ycf1
122609	G	A	ycf1
122620	G	A	ycf1
122703	A	C	ycf1
122737	G	A	ycf1
122778	C	T	ycf1
122884	C	A	ycf1
122900	G	A	ycf1
123104	G	T	ycf1

123155	T	C	ycf1
123156	A	C	ycf1
123276	G	A	ycf1
123282	C	T	ycf1
123303	C	T	ycf1
123305	G	A	ycf1
123392	T	G	ycf1
123408	C	A	ycf1
123428	G	A	ycf1
123532	A	G	ycf1
123564	A	C	ycf1
123573	C	T	ycf1
123606	A	G	ycf1
123623	T	G	ycf1
123628	G	A	ycf1
123664	G	A	ycf1
123670	C	T	ycf1
123685	A	C	ycf1
123719	G	A	ycf1
123761	C	G	ycf1
123769	T	C	ycf1
123786	T	G	ycf1
123828	A	C	ycf1
123877	T	G	ycf1
123917	C	T	ycf1
123925	T	G	ycf1
123926	T	G	ycf1
123929	T	C	ycf1
123959	T	G	ycf1
123969	T	C	ycf1
124034	C	T	ycf1
124112	A	C	ycf1
124131	C	A	ycf1
124192	G	T	ycf1
124230	A	G	ycf1
124248	A	C	ycf1
124355	T	G	ycf1
124370	G	A	ycf1
124374	A	C	ycf1
124389	T	G	ycf1
124423	A	G	ycf1
124442	G	A	ycf1
124478	G	T	ycf1
124500	G	A	ycf1

124511	C	T	ycf1
124539	A	G	ycf1
124602	G	C	ycf1
124630	T	C	ycf1
124721	T	C	ycf1
124727	A	C	ycf1
124869	T	G	ycf1
124899	G	A	ycf1
124915	G	A	ycf1
124934	A	G	ycf1
124938	T	G	ycf1
124952	C	T	ycf1
124977	G	A	ycf1
124989	T	A	ycf1
124997	C	A	ycf1
125060	C	T	ycf1
125073	T	C	ycf1
125196	G	A	ycf1
125205	C	T	ycf1
125214	G	A	ycf1
125261	T	C	ycf1
125355	T	C	ycf1
125487	C	T	ycf1
125496	G	A	ycf1
125554	T	A	ycf1
125567	C	T	ycf1
125624	A	G	ycf1
125627	G	T	ycf1
125629	A	C	ycf1
125634	G	T	ycf1
125822	C	A	ycf1
125825	G	T	ycf1
125841	A	G	ycf1
125951	A	G	ycf1
125954	A	G	ycf1
125978	T	C	ycf1
126000	A	C	ycf1
126050	T	G	ycf1
126078	A	C	ycf1
126082	C	T	ycf1
126105	A	G	ycf1
126115	A	C	ycf1
126143	C	T	ycf1
126174	C	T	ycf1

126175	A	C	ycf1
126209	C	T	ycf1
126210	T	C	ycf1
126222	A	C	ycf1
126253	A	G	ycf1
126260	G	A	ycf1
126329	T	G	ycf1
126337	C	A	ycf1
126339	A	G	ycf1
126351	A	G	ycf1
126397	C	T	ycf1
126413	T	G	ycf1
126458	T	C	ycf1
126476	A	C	ycf1
126480	T	C	ycf1
126601	G	A	ycf1
126767	A	G	ycf1
126786	T	G	ycf1
126798	A	C	ycf1
126846	G	T	ycf1
126887	T	C	ycf1
127008	G	A	ycf1
127095	T	A	ycf1
127116	C	A	ycf1
127186	A	C	ycf1
127482	A	C	ycf1

InDels identified in *P. obconica* subsp. *obconica* chloroplast genomes

InDels position	Ref	Alt
183	ATATTATATTAT	A
267	A	AAAGGAC
1614	A	ATAAATAGAAAT
1732	TA	T
2749	A	AAAG
3765	TTATAGGA	T
3836	GA	G,GAA
4039	TC	T
4489	GTAAATAA	G
4579	GA	G
4681	AAAATAT	A
4828	C	CAAATTGAAGAAATTGTCAGAATTTTTCTAGGATCAAT
4848	TTACTGTTATAGAACATA	T

	GGGGAGTAATGAATTGG TATGAATAGAGCAAAAG AGAAAATAAAAAGTTAG GGGAG	
4957	T	TATTACTCTAATTACTCTA TA
4959	T	TA
4962	C	CTCTATTATTCTATTATTTT TACTCTATTAT
4969	AT	A
5352	ATGGTTAAATAAATGAG ATTT	A
5393	TACTTAAAAAA	T
5397	T	TA
5749	A	ATTTT,ATT,AT,ATTTTT
6250	T	TATTATA
6667	T	TAAAGGAA
6911	TA	T
6942	T	TCAATATTAGACC
7983	C	CA
7992	AAGG	A
7993	AGG	A
7995	G	GTT,GATT
8282	GA	GAA,GAAAA,G
8625	CT	C,CTT
8679	G	GATAT
9778	TATTAC	T
9786	A	AT
9793	TA	T
9798	A	AGTAAT
9949	CT	C
10078	A	AT
11724	G	GA
12455	T	TATATATAA
12461	T	TAACAATAATAG
12474	C	CAATATATAA
12475	C	CA
12586	T	TCG
12588	GAA	GAAA,GA,G
13095	T	TTA
13113	C	CTAAATTCTTAAT,CTAAATTATTAAT,CTAAATTCTTAATTAAATTCTTAAT
13278	G	GGGTT

14203	A	AAC
14207	TCC	T
14215	TTTATCATTATTCCATATG AATAAAATGGAGATTAG GCCGAATCA	T
14301	AATTTATTATTTCTTACAT TTTTCTTTTGGTCAATCGT TGGTTTTATTAAATTGAA GGGGGAGTCGTTCT	A
14381	CTT	C,CT
15299	CAATTCTATA	C
16266	A	ATACATTATAGT,ATACAT ATAGT
22521	T	TAATACTTAA,TAATACTT AAAATACTTAA
22830	C	CCCTTT,CTTT,CCTTT
26670	T	TTTA
27301	A	AC
27464	TATTCATTAGTTATGTATA AATTTCTATTTTTTATGTC ATTAGGAAACCACAAA	T
27622	C	CT
27671	T	TATTCATTAGTTATGTATA AATTTCTATTTTTTATGTC ATTAGGAAACCACAAA
27709	T	TA
27948	GT	G
28066	C	CAATATTCTATTG
28216	C	CATATTCATAGAA
28751	AAACT	A
28869	T	TAATAA
29006	T	TATA
29010	T	TTATTAGA
29029	CTT	C
29034	C	CAG
29062	CT	C
29369	ATTTTT	A
29374	T	TA
29380	ATTTT	A
29852	T	TATAGTA
29937	AATGCATTCCTTCTCAAA TAAAGGCGCGATTGTGG GACGATCTTTATCTTTCTT TTATCCTC	A

30429	G	GA,GAAA,GAA
31008	ATT	AT,A
31016	T	TATCTAA
31018	T	TC
31077	T	TTCTATA
31084	TAATATATAA	T
31109	CTCATTTCTAATTCTCT	C
31124	CTTAATTTATATTAAATAAAAATAATAAATTAGAGTACGAAAAATACAATTTCGCCCGGAATCTAATAATTTGTCGAAAAAGAGGGAGTTTAATTCCA	C
31132	ATATTAAATAAAAATAATAAATTAGAGTAC	A
31146	T	TAAGA
31149	T	TAAATTAGAA,TTAGAA
31154	T	TAGAAAAATG
31161	CG	C
31164	AAAATACAAT	A
31176	CGCCCGGAA	C
31186	CTAATAATTTGTCGAAAAAGAGGGAGTTTAATTCCA	C
31260	T	TGCCTAGCG
31330	GAA	G
31331	AAATT	A
31333	ATTT	A,AT
31758	T	TCATTATAATGA
31762	T	TCTA
31764	T	TGACATTAG,TAATGACATTAG
32011	CTTT	CTTTT,CTT,CT,C
32126	TATTCC	T
32183	ACCAAGATTCATTTTTATTT	A
32224	A	ATATT
32474	C	CT,CTT
32634	AATAGGTT	A
35408	C	CTTT,CTTTTT,CT,CTT
35593	T	TA
35815	GA	G
36255	TTTAATA	T
36383	CATATATAT	C,CATAT

36400	CATAT	C
36584	AAAACAAAAAAAG	A
37278	TATACG	T
41850	ATTTTTT	A,ATTTTT
41865	TATATAAATTATTTC	T
41872	A	ATTATTTCTCTT,ATGATTT CTCTT
41879	C	CTCTTTTATTTT
42088	G	GCC
42134	TAGAACATATATTAAG	T
42239	T	TATCAATAG
42308	AGACAAATAT	A
42351	T	TTATAA,TTATAATATAA,T TC
42353	A	AATATTAT
42625	CTTTTT	C
43225	TC	T
43228	CGTTT	C
43743	A	AT
44124	A	ATT,AT,ATTT
44191	CA	C
44523	TAGATTTCA	T
44533	GATTTCAC	G
44641	GAAAAA	GA,GAAA,G,GAA
44695	TC	T
44871	AT	A
44928	T	TTAA
45313	ACTTTGATATTTGAACAG TTAGATCTTT	A
46321	CGATTATTATA	C
46348	A	AT
46418	G	GA
46427	AAGGG	A
46428	AGGGG	AGGG,AG,AAGGGGGG,A
46432	G	GAA
46452	C	CTT,CTTT,CT
46916	A	AGAAT
46926	CAT	C
46928	T	TTC
46930	A	AAGT,AT
47206	T	TTATTACTA
47208	A	ATTACTATATC
47210	T	TACTATATTACTATATCTA
47330	A	AAAAAG

	GGGGAACGACATCACAA	
47333	TGAGATCCTAATCTCAAA TCAAAA	G
47540	TC	T
47541	CAA	CA,C
47551	A	AGG,AAG
48026	AT	A
48120	T	TA
48133	G	GTTTT
48299	TGTTTTTTAA	T
48317	G	GACCCA
48516	TTTTTATCCATCTAAGTGC TTAGAGATCTACCCCATC TCTAAAATCGATGGGTAA ATTATATTC	T
48677	GGTTT	G
48684	T	TAACA
48744	T	TA
48767	ACC	ACCC,AC,A
48925	G	GA
48971	TC	T
48972	CTT	C,CT,CTTTTTT
48982	TG	T
49589	TTTGGAA	T
49591	TG	T
49594	AATTGG	A
49663	A	AT
49681	TCAC	T
49685	TTTC	T
51150	T	TGAATAAA
51429	T	TA
51742	G	GT
51793	T	TC
51860	T	TCTA
51862	T	TTTG
51870	T	TTTTATAGGGTTTA
52017	A	ATATAAAT
54985	C	CAATAAGAAATCGGGGT TTAGTTTAGTACTCCATTT TGTTGGTACCATTCACCC GAATCCAATTCAATTTAT TACTTAATTTAATAAGAT AAGTTAATTTTCAAATGA AACCAAACCCTTTTCAA

		AATATCAAGTGAATT,CAATAAGAAATCGGGGTTTAGTACTCCATTTT
55145	CT	CTT,C
55226	C	CA
55266	A	AG
55267	A	AGG,AGGG,AG
55350	C	CCAT
55391	A	AATATTAAT
55400	A	AT
57102	CAAGATTTAAAATTCAATAAAAT	C
57404	CT	C
57489	TAAA	TA,TAA,T
57501	T	TG
57609	G	GGAGTTA
57743	C	CTTACAGT
57746	A	ACAGTAAT
58715	CTTTATA	C
58722	T	TTTATACAATTATC
58933	CAGA	C
58934	AGAAG	A
58937	A	AGG
59514	T	TA
59627	A	ATTATTACAAGATATCT
59846	C	CTTTTT,CTTTTTT,CT
59955	T	TA
61064	A	ATT
61459	TACAAAA	T
61994	TA	T
63179	TAC	T
63180	AC	A
63181	C	CA
63308	TC	T
63310	CA	C
63320	AT	A
63381	GT	G
63519	T	TA
63540	GGTGTTGCA	G
63552	T	TTTCAACAC
63615	AT	ATTT,A,ATT
63717	AATG	A
63979	G	GA
65171	GGTGCCAC	G

65380	TGAAAAAAATAGA	T
65388	AT	A
65390	AGAGAAAAAAAT	A
65398	A	AAATAGAACTAAAG
65519	C	CTATGGAA
65537	GA	GAAA,G,GAA
66623	TA	T
66641	ATATATATAAT	A
66647	ATAATTATATATAATAT	A
66649	AAT	A
66655	ATATAATAT	A
67027	TC	T
67409	T	TTTCTCTTTCCTCCC
67653	GTATTACT	G
68074	T	TATATATAATAAAATAGTTA
68085	A	AAATATTTAATAAATACATTT
68477	TAGAACTAGA	T
68671	TA	T
68681	AATATAATAATC	A
69290	AT	A
69634	CTT	CT,C
69684	CT	CTTT,C
69846	G	GAAA,GA,GAAAA
70080	T	TAAAAAAATTAA
70473	T	TA
70668	ATATTTCT	A
70744	TA	T
70813	T	TGA
70910	TTTTTG	T
70915	G	GAAAAA
71873	C	CATTTAT
71917	T	TG
71981	T	TA
72007	CA	C
72014	A	AG
72016	GA	G
72262	AT	A
74265	A	AT
74372	TAAAAAAG	T
75381	CA	C
75391	AAT	A
75392	AT	A

75640	CAATAATA	C
75688	AT	A
75809	AT	ATT,A
77025	AAATAGAGAAACCCTAATTAGGGTTTCTCTATTTCACTTAATTTAAGATTG	A,AAATAATAGAGAAACCCTAATTAGGGTTTCTCTATTTCACTTAATTTAAGATTG
77053	C	CTAT
77286	CATGAT	C
77359	C	CAATAAAATAAATA
77377	T	TA
77379	C	CTCTGGAAAA
77380	T	TCTGGAAAAG
77413	A	ATTTAATTTATAT
77460	T	TGTAG
78101	ATATTACGAC	A
78164	T	TA
78165	AATCAATTAAATT	A
78491	A	ATTTTTT
79341	A	AT
79840	T	TC
79841	C	CT
79992	T	TATTTTTA
80313	AT	A
80345	T	TA
80399	GT	G,GTT
80931	TGA	T,TA
81487	C	CA
82022	G	GAA,GA
82047	CATTTGAATTGATATAATTGATATATATATATATTTAATGAATAATAGATGAAATCGCGGTATTCTTTGACATTGAATCTAAATCCTATTAGTGTTTTGTATACCCTGTTTGGGTATTTTGGATATATAATTAAACCTATTAAACATAGATTTCTATTTA	C
82052	GAATTGATAT	G
82057	GATATAATTGAT	G
82563	T	TA
82747	ATTTTTTT	ATTTTT,ATTTTTT,A
82749	T	TC
82904	GT	G,GTT

83840	AATTCATCTTCAT	A
111415	T	TTCTTACTTATTTTTTTAT GTG
111598	GAA	G
111602	TAAAACAC	T
111610	TAAA	TA,TAA,T
111653	TA	T
111656	GTTAGCCTAATGAAATAA TAACTCCTCTTTTTCTTTA TTATTTCATTTATTCAAAA TAATTAACTAGTTCATTTT GAAATTGA	G
111766	CG	C
111817	G	GAAACTA
111825	A	AAC
111826	T	TAAAC
111910	T	TA
111911	ATT	A,AT
111917	T	TA
111925	G	GTTT,GTT,GTTTT,GT
112377	T	TA
112520	GAAACCTTCAAATTACTA TTCATTTGAATTCATCAA TTTTATTCTTTCTTAAAAC TTAAAAAAAA	G
112574	CT	C
112576	TA	T
112747	TA	T,TAA
112761	AAATAG	A
113382	C	CACCA
113388	A	AAAAGAT
113392	TTTTCCTTTGG	T
113845	T	TATAAAAA
113847	TA	T
113988	T	TGTACAGC,TGTACGGC
113990	T	TACAGCGTACAGCGA
117464	CTAAT	C
117469	CAATCA	C
118809	G	GATATT
118823	TTAATA	T
119715	TAAAAATGGATTCAATTC ATTTCATTTTTTTACTAGA AACG	T
120332	CTGTTGT	C

121542	GT	G
121611	TTTTC	T
121616	A	AAAAG
122115	GAAATAATGGATATCCCA TTACATTAA	G
122127	AT	A
122129	CCCATTACATTAAAAATA ATGGATAT	C
125519	C	CTTTTTTT
125526	T	TCTTTTCTC
126551	TTATTAA	T
126804	AAGTATTT	A
126815	A	AT
