# Supplementary material for: The Complete Chloroplast Genomes of Primula obconica Provide Insight That Neither Species nor Natural Section Represent Monophyletic Taxa in Primula (Primulaceae)
Source: Genes (Basel). 2022 Mar 23;13(4):567. doi: 10.3390/genes13040567 (PMC9030805; doi:10.3390/genes13040567)
Supplement: Supplementary file 1 [file genes-13-00567-s001.zip › Supplemental Table S6.pdf]

Table S6. SNP annotation based on 16 chloroplast genomes of *P. obconica* subsp. *obconica* using SnpEff

Type	Count	Percent	Ts/Tv (transitions / transversions)
Total SNPs	1,915		
Total possible effects	21,371		
Downstream gene variant	9,737	45.549%	
Intergenic region	757	3.541%	
Intron variant	1,192	5.576%	
Missense variant	360	1.684%	
Splice region variant	6	0.028%	1.1667
Stop gained	2	0.009%	
Stop lost	2	0.009%	
Stop retained variant	1	0.005%	
Synonymous variant	361	1.689%	
Upstream gene variant	8,959	41.91%	
