# Supplementary material for: The Complete Chloroplast Genomes of Primula obconica Provide Insight That Neither Species nor Natural Section Represent Monophyletic Taxa in Primula (Primulaceae)
Source: Genes (Basel). 2022 Mar 23;13(4):567. doi: 10.3390/genes13040567 (PMC9030805; doi:10.3390/genes13040567)
Supplement: Supplementary file 1 [file genes-13-00567-s001.zip › Supplemental Table S7.pdf]

Table S7. List the SSRs that shared by *P. obconica* subsp. *obconica*.

Repeat motif									Location
YN1	HBD	HNL	HND	HBE	ZP2	ZP1	SCD	SCB	
(T)10\(A)10	(A)10\(T)12	(T)10	(T)10	(T)10	(T)13	(T)10	(A)10	(A)10	rps16
(T)11	(T)11	(T)11	(T)11	(T)11	(T)11	(T)11	(T)10\(T)11	(T)11	rpoC2
(T)10	(T)11	(T)11	(T)11	(T)11	(T)11	(T)11	(T)10	(T)10	rpoB
(AATA)3	(AATA)3	(AATA)3	(AATA)3	(T)10\(AATA)3	(AATA)3	(T)10\(TATAA)3\(AATA)3	(AATA)3	(AATA)3	psaA-ycf3
(A)10	(A)10\(T)10	(A)11\(T)10	(A)11\(T)10	(A)10\(T)10	(A)11, (G)10\(T)10	(A)11\(T)11	(A)12	(A)11	rps4-trnT
(T)10	(T)10	(T)10	(T)10	(T)10	(T)10	(T)10	(T)10	(T)10	atpB
(A)10\(A)10	(A)10\(T)12	(A)10\(T)12	(A)10\(T)12	(A)10\(A)11\(T)12	(A)10\(T)11	A)11\(A)10\(T)12	(A)10\(T)10	(A)10	petA-psbJ
(TA)7	(TA)7	(TA)7	(TA)7	(TA)7	(TA)7	(TA)7	(TA)9	(TA)6	trnW-trnP
(A)10	(A)10	(A)12	(A)12	(A)11	(A)11\(T)10	(A)10	(A)11	(A)11	petB
(AT)7\(TTTC)3	(A)11\(AT)7	(A)10\(AT)7\(T)10	(A)10\(AT)7\(T)10	(AT)6	(T)16	(A)10\(AT)7	(A)10\(AT)7\(TTTC)3\(T)10	(A)11\(TTTC)3	rpl16
(A)13	(A)13	(A)13	(A)13	(A)13	(A)13	(A)13	(A)13	(A)13	trnN-ndhF
(A)11	(A)11	(A)11\(A)11	(A)11\(A)11	(A)12\(A)11	(A)12\(A)10	(T)10\(A)11\(A)10\	(A)11	(A)11	rpl32-trnL
(AATA)3	(AATA)3	(AATA)3	(AATA)3	(AATA)3	(AATA)3\(A)10	(AATA)3	(AATA)3	(AATA)3	ndhD
(GAAA)3	(GAAA)3	(GAAA)3	(GAAA)3	(GAAA)3	(GAAA)3	(GAAA)3	(GAAA)3	(GAAA)3	ndhA
(T)11 \(T)11 \(T)11\(T)12\(T)13	(T)11 \(T)11 \(T)11 \(T)11\(T)12\(T)13	(T)11 \(T)11 \(T)11\(T)12\(T)13	(T)11 \(T)11 \(T)11\(T)12\(T)13	(T)11 \(T)11 \(T)11 \(T)11\(T)12\(T)13	(T)11 \(T)11 \(T)11 \(T)11\(T)12\(T)13	(T)11 \(T)11 \(T)11\(T)10\(T)12\(T)13	(T)11 \(T)11 \(T)11\(T)12\(T)13	(T)11 \(T)11\(T)12\(T)13	ycf1
