# Supplementary figures and images for: The Complete Chloroplast Genomes of Primula obconica Provide Insight That Neither Species nor Natural Section Represent Monophyletic Taxa in Primula (Primulaceae)
Source: Genes (Basel). 2022 Mar 23;13(4):567. doi: 10.3390/genes13040567 (PMC9030805; doi:10.3390/genes13040567)

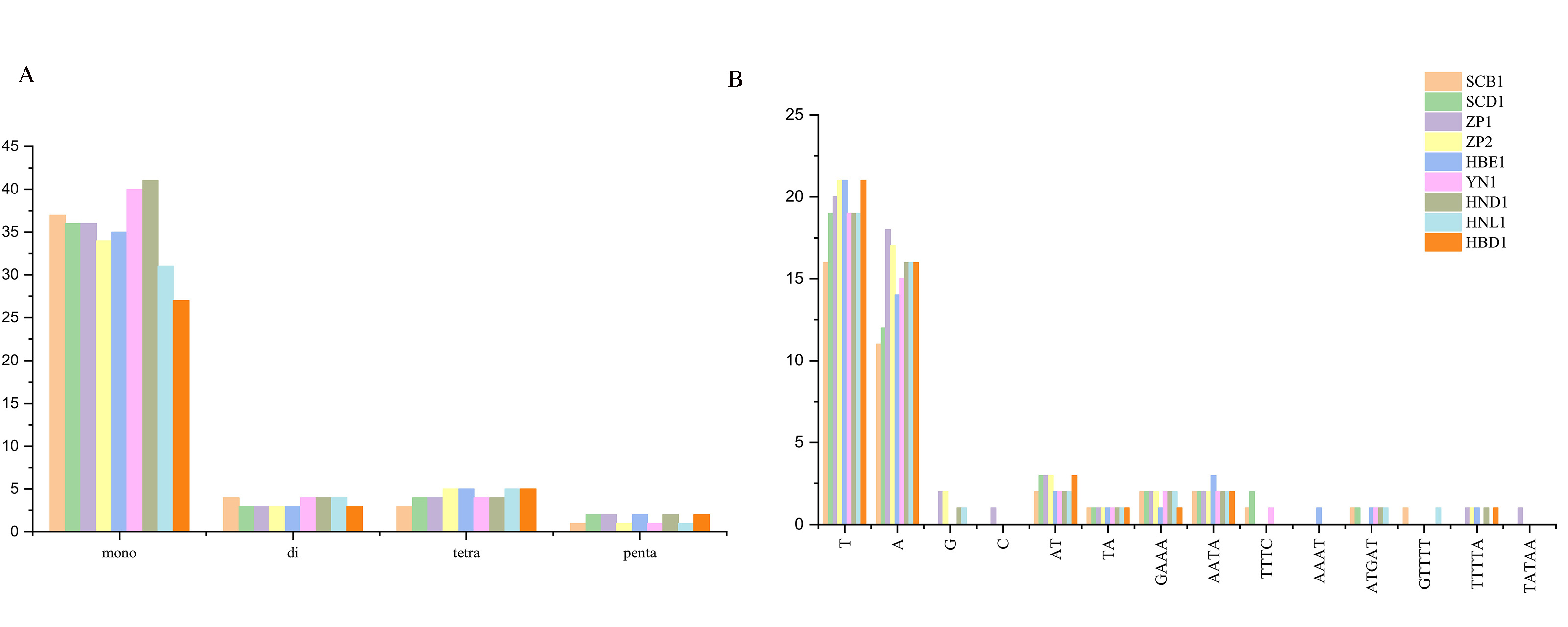

Supplement: Supplementary file 1 [file genes-13-00567-s001.zip › Supplemental Figure S1.jpg]

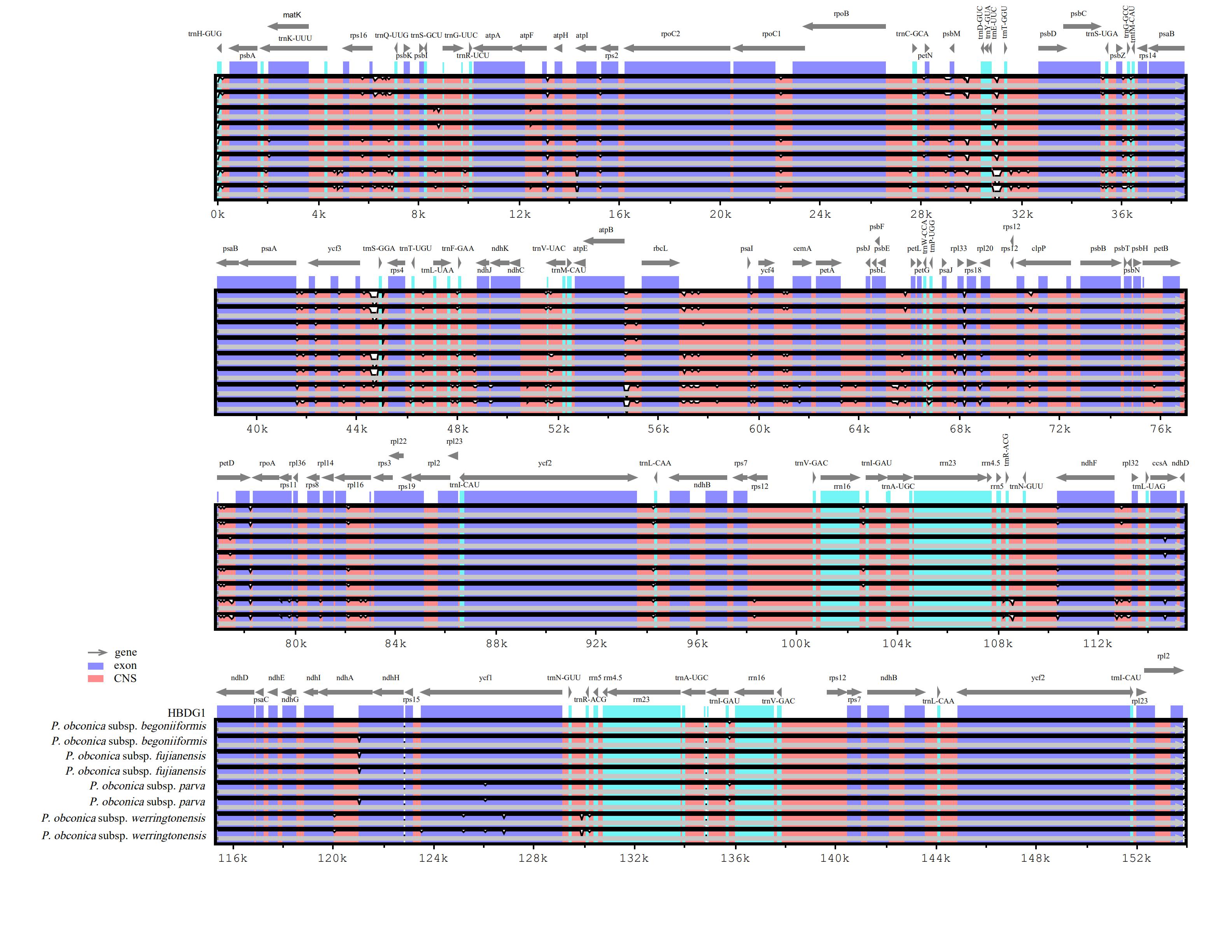

Supplement: Supplementary file 1 [file genes-13-00567-s001.zip › Supplemental Figure S2.jpg]
